# Metabolomic Response of Early-Stage Wheat (*Triticum aestivum*) to Surfactant-Aided Foliar Application of Copper Hydroxide and Molybdenum Trioxide Nanoparticles

**DOI:** 10.3390/nano11113073

**Published:** 2021-11-15

**Authors:** Xiangning Huang, Arturo A. Keller

**Affiliations:** 1 Center for Environmental Implications of Nanotechnology, University of California, Santa Barbara, CA 93106, USA; xiangningh@ucsb.edu; 2 Bren School of Environmental Science and Management, University of California, Santa Barbara, CA 93106, USA

**Keywords:** surfactants, nanomaterials, nanoagrochemicals, metabolomics, wheat

## Abstract

Surfactants are commonly used in foliar applications to enhance interactions of active ingredients with plant leaves. We employed metabolomics to understand the effects of Triton^TM^ X-100 surfactant (SA) and nanomaterials (NMs) on wheat (*Triticum aestivum*) at the molecular level. Leaves of three-week-old wheat seedlings were exposed to deionized water (DI), surfactant solution (SA), NMs-surfactant suspensions (Cu(OH)_2_ NMs and MoO_3_ NMs), and ionic-surfactant solutions (Cu IONs and Mo IONs). Wheat leaves and roots were evaluated via physiological, nutrient distribution, and targeted metabolomics analyses. SA had no impact on plant physiological parameters, however, 30+ dysregulated metabolites and 15+ perturbed metabolomic pathways were identified in wheat leaves and roots. Cu(OH)_2_ NMs resulted in an accumulation of 649.8 μg/g Cu in leaves; even with minimal Cu translocation, levels of 27 metabolites were significantly changed in roots. Due to the low dissolution of Cu(OH)_2_ NMs in SA, the low concentration of Cu IONs induced minimal plant response. In contrast, given the substantial dissolution of MoO_3_ NMs (35.8%), the corresponding high levels of Mo IONs resulted in significant metabolite reprogramming (30+ metabolites dysregulated). Aspartic acid, proline, chlorogenic acid, adenosine, ascorbic acid, phenylalanine, and lysine were significantly upregulated for MoO_3_ NMs, yet downregulated under Mo IONs condition. Surprisingly, Cu(OH)_2_ NMs stimulated wheat plant tissues more than MoO_3_ NMs. The glyoxylate/dicarboxylate metabolism (in leaves) and valine/leucine/isoleucine biosynthesis (in roots) uniquely responded to Cu(OH)_2_ NMs. Findings from this study provide novel insights on the use of surfactants to enhance the foliar application of nanoagrochemicals.

## 1. Synopsis

Surfactants enhance the delivery of NMs via foliar exposure, with minimal physiological effects. However, surfactants do elicit metabolic reprogramming. NM size, zeta potential, dissolution rate, adhesion of surfactant and NMs to plant leaves, and translocation are all important factors during NMs foliar applications. Plant molecular responses to surfactant-aided NM application should be taken into account to maximize the benefit of nanoagrochemicals and reduce negative effects.

## 2. Introduction

Nanosized materials (NMs) are increasingly being considered to improve agricultural sustainability. Nanofertilizers (e.g., Ca, Fe_2_O_3_, MgO, P, CuO, MnO, MoS_2_, and ZnO) [1,2,3] and nanopesticides (e.g., Ag, single-walled carbon nanotubes (SWCNTs), CeO_2_, Cu(OH)_2_, MgO, Mg(OH)_2_, SiO_2_, and TiO_2_) [2,4,5,6] have been investigated in previous studies. Some NMs (e.g., TiO_2_, SiO_2_, and SWCNTs) have also been considered to alleviate salt/drought stress or enhance plant photosynthesis [7,8,9]. Due to interactions between NMs or their dissolved ions with soil constituents and root exudates [10,11], they are not fully taken up by the plant from the soil, resulting in inefficient delivery of the active ingredient(s). Foliar delivery provides an efficient and scalable approach for direct interaction between NMs and plants [12]. However, the aggregation and dissolution of NMs, environmental conditions (e.g., rain and wind), as well as various leaf surface characteristics can substantially affect the efficiency of foliar applications [13,14]. In particular, the abundant cuticular waxes and trichomes (hairs) on wheat (*Triticum aestivum*) leaf surfaces make them extremely difficult to wet, with little or no water adhesion [15]. To improve the efficiency of foliar application, adjuvants (e.g., humectants, oils, pH buffers, and surfactants) are often employed to improve the wettability of leaf surfaces and prevent off-target drift [16]. We hypothesize that the combination of nanoagrochemicals with surfactants can improve the delivery of the active ingredients via foliar applications, particularly for plants with hydrophobic or superhydrophobic leaf surface properties. The main objectives of this study were to (1) employ a surfactant to improve delivery of NMs to plants; (2) determine whether the surfactant had any effect on the perturbation of target crop metabolic pathways; (3) evaluate the delivery of nano-scale active ingredients through foliar application; and (4) determine the metabolic response of plants to surfactant-enhanced NMs foliar exposure.

Surfactants are often used to lower the surface tension between droplets and leaf surfaces, as well as to improve penetration of active ingredients through plant cuticles [17]. Surfactants are usually classified as anionic, cationic, non-ionic, and amphoteric [18]. Non-ionic surfactants are widely used in many agrochemical formulations [17]. In the current work, Triton^TM^ 100-X (polyethylene glycol tert-octylphenyl ether, a non-ionic surfactant) was added into NMs suspensions during the wheat foliar applications. Past work has shown that Triton^TM^ 100-X not only enhances the wettability of wheat leaf surfaces, but also promotes nutrient uptake through foliar applications [15,19,20]. Compared with two other surfactants (sodium dodecyl-sulfate and dodecyltrimethylammonium bromide), Triton^TM^ 100-X solution (>1 × 10^−^^5^ mol L^−^^1^) resulted in pronounced wettability and considerably decreased the contact angle (<90°) of water drops on wheat leaves [20]. In another study, two commercial surfactants (3 g/L LI 700^@^ and 1 g/L Agral^@^), two pure surfactants (1 g/L Genapol^@^ X-080 and 1 g/L Triton^TM^ 100-X), and one humectant (1 g/L glycerol) were employed to assist phosphorous (P) translocation in wheat through foliar applications [15]. Except for glycerol, P uptake was greater than 70% for all surfactants; Triton^TM^ 100-X resulted in 82.4–83.5% increased P uptake during early tillering and flag leaf emergence periods. Past agricultural surfactant-related studies have mostly focused on interactions between surfactant and leaf surfaces [19,20,21], the development of new surfactants formulations [17,22], and applications of surfactants with conventional fertilizers/pesticides [15,23,24,25]. Few studies have considered the use of surfactant during foliar applications of NMs [26,27].

Many factors can affect the effectiveness of NMs foliar application processes, including NM properties (e.g., size [28,29,30], concentration [31,32,33], zeta potential [30], response duration [31], and coating [29,30]), leaf surface characteristics (e.g., wax thickness, pores, trichomes, and epidermis maturity) [34,35], NM uptake pathways (i.e., stomata [32,36,37] vs. cuticle [29,30]), and environmental factors [2,27,28,29]. For example, Avellan et al., (2019) investigated the effects of Au NMs sizes and coatings for wheat foliar exposure without the assistance of surfactants [30]. Regardless of the tested conditions, smaller Au NMs (3 nm) had a stronger adhesion to leaf surfaces, and 10–25% of the Au was transported to wheat roots after the rinsing process. Au NMs coated with polyvinylpyrrolidone (PVP) appeared in the mesophyll (cuticular pathway) and moved further through the plant vasculature. However, similar-sized Au NMs with a citrate coating were not present. In another short-term exposure study (24 h), wheat leaves exposed to zinc salts containing 0.05 wt% of Tween20 resulted in about six times greater Zn content in wheat leaves than the ZnO NMs-Tween20 foliar exposure. However, no significantly Zn content differences were found among ZnO NMs-Tween20 suspensions with different coatings [29]. The authors proposed that ionic adsorption was likely the dominant mechanism for passing through the cuticular pathway. Most of the above-mentioned studies either did not include surfactants, or simply employed surfactant as the wetting agent, without analyzing the cellular level plant response. To date, little is known about the metabolomics of applying surfactants on crop plants.

Metabolomics is increasingly being employed to study plant molecular responses/mechanisms to different stimulus (e.g., NMs) [4,38,39,40,41,42,43,44,45,46,47,48,49,50]. Given the extended use of the commercial nanopesticide Kocide 3000 (active ingredient: Cu(OH)_2_ NMs), the effects of Cu(OH)_2_ NMs on basil (*Ocimum basilicum*) [4], cucumber (*Cucumis Sativus*) [44,46], maize (*Zea mays*) [44,45], and lettuce (*Lactuca Sativa*) [47] have been explored. For example, Zhao et al., (2017) conducted a one-week Cu(OH)_2_ NMs foliar spray on corn (*Zea mays*) that systematically studied plant cellular responses [44,45]. The general plant response was dose-dependent, with a higher dose (100 mg per plant) significantly upregulating 4-hydroxycinnamic acid (2.22 fold), myo-inositol (1.50 fold), tyrosine (1.39 fold), phenylalanine (1.24 fold), and total phenolic content (1.17 fold) in maize leaves [45]. The biological pathway analysis revealed that the most significantly disturbed pathway was inositol phosphate metabolism [44].

In the current study, the molecular response to foliar application of MoO_3_ NMs was also evaluated. Molybdenum (Mo) is an essential micronutrient, often involved in reductive and oxidative reactions via specific plant enzymes [51]. Mo also plays an important role in N fixation, nitrate reduction, and amino acid and protein biosynthesis processes [52]. Excess exposure to Mo NMs and Mo^6+^ can inhibit root growth/elongation, prolong seed germination, increase nitrate reductase, and cause oxidative imbalance [53,54,55,56,57]. Recently we demonstrated that corn and wheat exposed to MoO_3_ NMs through a three-week root exposure (at 200 and 1000 mg/kg Mo levels) resulted in a significant response in plant roots and leaves at the molecular level [49]. Although the physiological data showed that the MoO_3_ NMs had a more severe impact on corn roots and above-ground plant tissues, 53 dysregulated metabolites were found in wheat leaves compared with 21 in corn leaves. Furthermore, TCA cycle, amino acid metabolism, and pyrimidines metabolism were perturbed in wheat leaves, but not in corn. To our knowledge, there have been no studies regarding surfactant-enhanced foliar application of Cu(OH)_2_/MoO_3_ NMs and the subsequent metabolomic alterations. Furthermore, due to the natural loss of NMs during the spraying process, to date, most foliar application studies have been qualitative rather than quantitative.

In this study, three-week-old wheat seedlings were exposed to deionized water (DI) only, surfactant only solution, NM-surfactant suspensions, and ionic-surfactant solutions through one-week foliar exposures. Wheat was selected since it is an important global crop, which requires crop protection from pests. Wheat responses to the surfactant (without active ingredients) were evaluated separately in order to determine how the surfactant would affect plant response at the molecular level. At the end of the exposure duration, plants were harvested and separated into wheat roots/leaves and changes in physiological parameters (e.g., dry biomass) were recorded. Target metals (e.g., Cu and Mo), along with other macro and micronutrient levels were analyzed to track metal translocation and distribution changes. Targeted metabolites, including amino acids, antioxidants, fatty acids, nucleobase/side/tide, organic acids/phenolics, and sugar/alcohols were measured and dysregulated metabolites were identified under each exposure condition. Furthermore, a perturbed metabolic pathways analysis was also conducted and the distinct effects of the two NMs (e.g., Cu(OH)_2_ NMs and MoO_3_ NMs) on wheat growth was evaluated. This work sheds insights into the effects of a surfactant on plant growth and the importance of enhancing foliar application conditions for NMs.

## 3. Materials and Methods

### 3.1. Characterization and Stability of Cu(OH)_2_ and MoO_3_ NMs

Cu(OH)_2_ NMs (99.5% purity, US3078) and MoO_3_ NMs (99.94% purity, US3330) were purchased from U.S. Research Nanomaterials Inc. (Houston, TX, USA). The original powders have been fully characterized elsewhere [11]. Briefly, the Cu(OH)_2_ NMs are nanowires (diameter: 50 nm and length: 2–5 μm) and the MoO_3_ NMs are spheres (diameter: 13–80 nm) (Figure S1). Both ENMs have an orthorhombic crystalline structure as confirmed by X-ray diffraction (XRD) analysis, and no carbon-based coatings were detected based on X-ray photoelectron spectrometry (XPS) measurements.

Triton^TM^ X-100 (BioXtra, Product No. T9284) was obtained from Sigma Aldrich (St. Louis, MO, USA). Based on the information provided by the manufacturer, the critical micelle concentration (CMC) is 0.2–0.9 mM (20–25 °C). A droplet dispersion test was conducted by depositing a 5 μL droplet of Triton^TM^ X-100 solutions on three-week-old wheat leaf surfaces (Figure S2). The optimum application level was 200 mg/L (0.32 mM), with adequate droplet spreading without dripping off. The applied concentration of Triton^TM^ X-100 solution in the current work is lower than previous foliar application studies, where 500–2000 mg/L of Triton^TM^ X-100 were employed [15,23,26]. The influence of the surfactant on NM zeta potential, hydrodynamic diameter, and dissolution rate was measured for each NM [11,58]. The tests were conducted in 50 mL metal-free polypropylene tubes that contained 200 mg/L Triton^TM^ X-100 with 100 mg/L Cu(OH)_2_ or 100 mg/L MoO_3_ NMs (as metal content) suspensions. After spiking the corresponding amount of NMs, the tubes were sonicated for 20 min and the hydrodynamic diameter and surface charge (zeta potential) were measured via dynamic light scattering (Zetasizer Nano ZS, Malvern, Westborough, MA, USA). For the dissolution test, 2 mL samples were withdrawn at 0 h and 5 h, and centrifuged in Amicon Ultra 3 kDa MWCO tubes (Sigma-Aldrich, St. Louis, MO, USA ) at 3000 rpm for 30 min [11,59]. Then the filtrate was acidified to 10 mL with 2% HNO_3_ (SCP Science, Product No. 250038175) for inductively coupled plasma-mass spectrometry (ICP-MS) (Agilent 7900, Agilent Technologies, Santa Clara, CA, USA) analysis. Three replicates were utilized for all analyses.

### 3.2. Wheat Growth and Leaf Exposure Assay

Wheat (*Triticum aestivum* ‘Red Fife’) seeds were selected for the current study. Before germination, seeds were immersed in a 1% sodium hypochlorite solution (Supelco. Product No. XX0637-76) for 10 min, rinsed well with deionized water, and soaked for 24 h. Four seeds were germinated in each pot (40 g vermiculite/pot) containing water-saturated vermiculite. Five days later, seedlings were transplanted to a new pot with two plants per pot. Growth conditions were 150 µmol·m^−2^·s^−1^ light intensity (as daylight white) for 16 h daily, the temperature was set at 22 °C, and relative humidity was 60%. A 10% Hoagland solution was used for watering purposes to ensure adequate nutrients for plant growth [49]. The water content in the pots was maintained between 70 and 90%.

Foliar exposure was initiated at the beginning of the fourth week and the exposure duration was 7 days. A preliminary test showed that without adding a surfactant, immersing wheat leaves in a 100 mg/L MoO_3_ NMs suspension resulted in little Mo accumulated on wheat leaves, with minimal translocation to roots (Figure S3). This finding confirmed the crucial role of the surfactant during the wheat foliar application process [15,19,20]. For the leaf exposure test, the second and third true leaves were exposed to metal-surfactant suspensions (two times per day) using a multichannel micropipette (2.54 ± 0.30 μL/drop, eight channels). The treatments were as follows: DI—deionized water only; SA—200 mg/L Triton^TM^ X-100 solution; Cu(OH)_2_ NMs—100 mg/L Cu(OH)_2_ NMs (as Cu content) in SA; Cu IONs—0.1 mg/L CuSO_4_·5H_2_O (as Cu content) in SA; MoO_3_ NMs—100 mg/L MoO_3_ NMs (as Mo content) in SA; and Mo IONs—35 mg/L Na_2_MoO_4_·2H_2_O (as Mo content) in SA. Eight replicates were employed for each experimental condition. At the end of the exposure period, all plants were harvested, well rinsed [14]. freeze-dried, and weighted. All freeze-dried plant tissues were stored at −80 °C for further analyses.

### 3.3. Plant Tissue Digestions and Nutrient Measurements

Freeze-dried plant tissues (i.e., roots and leaves) were cut into small pieces and transferred into 50 mL digestion tubes. The digestion process was conducted in an SCP Science DigiPREP hot block digestion system. First, 2 mL of plasma pure HNO_3_ was added into the tube and heated at 115 °C for 20 min. Then, 8 mL of H_2_O_2_ reagent (Thermo Scientific™, Product No. H325-4) was added into the system and held at 115 °C for another 60 min [45,49]. When the digestion process was complete, all samples were diluted to the 50 mL mark for nutrient analysis. In addition to the targeted metals (i.e., Cu and Mo), four macronutrients (Ca, K, Mg, and P) and three additional micronutrients (Fe, Mn, and Zn) were quantified through ICP-MS analysis. The dried biomass and nutrient data were analyzed by using the statistical package SPSS (Social Sciences, 22.0). More specifically, a one-way analysis of variance (ANOVA) was performed followed by a Tukey-HSD test (*p* value = 0.05).

### 3.4. Plant Tissue Extractions and Targeted Metabolites Measurements

A total of 82 targeted metabolites (Table S1) were selected from previous studies, which showed good sensitivities during plant (corn, cucumber, soybean, and wheat) exposures to various NMs (Ag, Cd, Cu, and Mo) [49,59,60,61,62]. Briefly, the freeze-dried plant samples were finely ground by using pestle and mortar with liquid nitrogen. Then, around 10 mg of sample was weighed and transferred into a 2 mL Eppendorf microcentrifuge tube containing 1.2 mL of 80% methanol (Sigma-Aldrich, Product No. 646377-4L) and 2% formic acid (Supelco. Product No. 00940-50ML) [60,61]. The tubes were first vortexed for 20 min, then sonicated for 20 min, and finally centrifuged at 2 × 10^4^ g for another 20 min. The supernatant was analyzed through liquid chromatography—triple quadrupole mass spectrometry (LC-MS/MS) analysis (Agilent 1260, Agilent Technologies, Santa Clara, CA, USA) (see SI for more details).

Metabolite data was log-transformed and auto-scaled before further analyses using MetaboAnalyst 5.0 (MetaboAnalyst, CA, https://www.metaboanalyst.ca/). For the statistical analysis, a one-way ANOVA followed by a Fisher’s least significant difference method (Fisher’s LSD, *p* value set as 0.05) was conducted to identify the significantly altered metabolites. In addition, the PLS-DA method was applied. PLS-DA has often been used to maximize the metabolite cluster differences between treatment groups from the previous metabolomics studies [39,41,44,59]. From the PLS-DA test results, variables with importance in the projection (VIP) values greater than 1 were also considered as the featured metabolites. For the metabolomic pathway analysis, the perturbed pathways with an impact value threshold greater than 0.1 (*p* value threshold was 0.05) were considered [41].

## 4. Results and Discussion

### 4.1. NM Characterization and Dissolution

**Figure 1:**
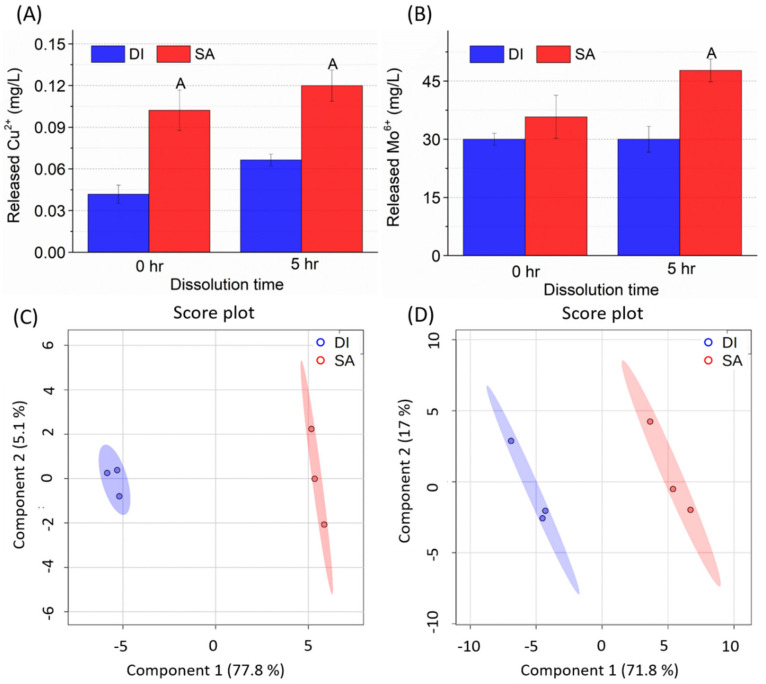
Metal ions released from (**A**) 100 mg/L Cu(OH)_2_ NMs and (**B**) 100 mg/L MoO_3_ NMs in DI water (DI) and surfactant containing suspensions (SA). PLS-DA score plots of overall metabolites in wheat (**C**) leaves and (**D**) roots after one-week foliar exposure to DI and SA solutions. Three replicates were used under each condition.

Cu(OH)_2_ and MoO_3_ NMs were characterized in the 200 mg/L Triton^TM^ X-100 suspension (SA) in terms of hydrodynamic diameter, zeta potential (ξ), and dissolution rate. Compared with their behavior in DI water, placing the NMs in SA resulted in slightly larger (but not significant) hydrodynamic diameters (Table S2). However, in SA ξ increased significantly for Cu(OH)_2_ NMs (4.84 to 15.9 mV) and MoO_3_ NMs (−65.0 to −51.9 mV) compared to in DI water. In general, the addition of surfactant accelerated NM dissolution (Figure 1A,B). More specifically, the overall dissolved amount of Cu(OH)_2_ NMs was quite small (0.1%) in SA. In contrast, 35.8% of MoO_3_ NMs was dissolved in SA with 33.5% more Mo^6+^ ions released after 5 h. This demonstrates the importance of refreshing NM-SA suspensions after each foliar exposure to maintain a similar ionic composition. Based on the NM dissolution results, the corresponding ionic exposure doses were determined as 0.1 mg/L of CuSO_4_ · 5H_2_O as Cu (Cu IONs) and 35 mg/L of Na_2_MoO_4_·2H_2_O as Mo (Mo IONs).

### 4.2. Plant Responses to the Surfactant Solution

We examined the wheat responses to foliar application of 200 mg/L Triton^TM^ X-100 solutions (SA) by quantifying the plant dry biomass, nutrient distribution, and metabolomic profile alteration. Compared with DI water foliar application, the surfactant solution did not significantly change the dry biomass, nor the majority of the nutrient content (i.e., Mg, P, K, Ca, Mn, Fe, Cu, and Zn) in wheat (Figure S4 and Table S3). However, a significant decrease in Mo content (59.1%) was observed in wheat leaves. Mo is an essential component of nitrate reductase and N-fixing enzyme nitrogenase; decreasing Mo could potentially impact N metabolism in wheat leaves [63]. In addition, the metabolomic profile showed a clear separation between DI and SA treatments, which could be explained by the total variance along component 1 in wheat leaves (77.8%) and roots (71.8%) (Figure 1C,D).

**Figure 2:**
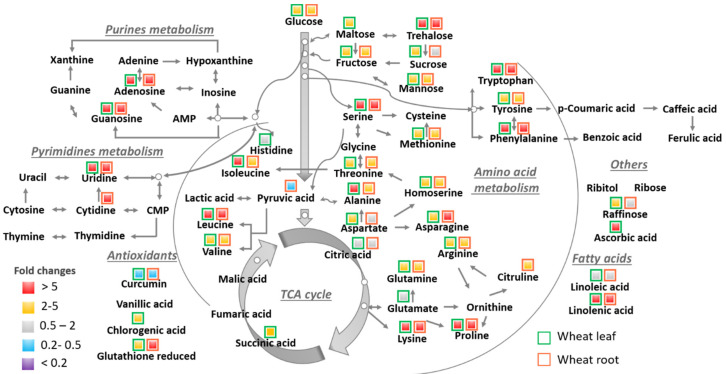
Significant metabolic pathway changes in wheat after one-week foliar exposure to surfactant solution. The color scale indicates the fold changes compared with DI water. The border of the box indicates whether the metabolite changes happened in wheat leaves (green) or roots (red).

When wheat leaves were exposed to SA, 37 metabolites were significantly altered in wheat leaves and 34 dysregulated metabolites were discovered in wheat roots (Table S4). Furthermore, more than 85% of these dysregulated metabolites were significantly upregulated (with fold change > 2) (Figure 2). The most significantly altered metabolites (with fold change > 5) were certain amino acids (leucine, lysine, phenylalanine, proline, serine, and tryptophan), fatty acids (linolenic acid), nucleobase/side/tide (adenosine, guanosine, and uridine), and sugar/sugar alcohols (trehalose). Amino acids are synthesized from TCA cycle intermediates; the increased levels indicate some favoring of N metabolism over C metabolism [44]. As one of the most abundant fatty acids in plant membrane lipids, the increased concentration of linolenic acid suggests potential membrane lipid peroxidation as a defense mechanism to abiotic stress [64]. The significant changes in the abundance of adenosine, guanosine, and uridine indicate the perturbation of pyrimidine and purine metabolism. Even though the role of trehalose remains unclear, a study has shown that this carbohydrate may serve to preserve the plant’s cellular integrity under stress conditions [65]. Foliar exposure to SA resulted in 17 perturbed metabolomic pathways in wheat leaves and 15 metabolomic pathways were significantly altered in roots (Table S5). Arginine biosynthesis and arginine/proline metabolism were only disturbed in wheat leaves, which involve the uniquely dysregulated metabolite from wheat leaves—glutamate. Glutamate plays a central role in the synthesis of γ-aminobutyric acid (GABA), arginine, and proline, and regulates ammonium assimilation [66]. The increased glutamate level may indicate a response to excess ROS [60]. On the other hand, pyruvic acid was only significantly downregulated in wheat roots (with fold change < 0.5), which contributed to the perturbation of valine, leucine, and isoleucine metabolism. As a precursor for valine, leucine, and isoleucine [67], pyruvic acid is also used as a substrate for the TCA cycle. The depletion of pyruvic acid indicates possible redistribution of N and C metabolism.

### 4.3. Wheat Leaf Responses to Metal-Surfactant Suspensions

With the assistance of the surfactant (200 mg/L Triton^TM^ X-100), the NMs and ionic salts were able to adhere to wheat leaf surfaces more effectively, resulting in a significant increase in target metals (i.e., Cu and Mo) found on wheat leaves (Figure S6A,B). Overall, there were no significant changes in wheat leaf dry biomass for all treatments (Figure S5A). Foliar exposure to Cu(OH)_2_ NMs resulted in an average of 649.8 μg/g Cu detected in wheat leaves, which is well above the Cu content found in other treatments. In addition, exposure to Cu(OH)_2_ NMs also significantly increased Zn content by almost 54% in wheat leaves (Table S6). Previous Cu(OH)_2_ NMs foliar exposure-related studies often found no significant changes in Zn content [4,45,46], however, one study did observe increased Zn in vascular tissues of lettuce (*Lactuca sativa*) [47]. Zn is an essential micronutrient for plant growth and is involved in several essential processes. Plants with high Zn efficiency exhibit healthy growth status and high yield [68]. Exposure to Cu IONs had a minimal effect due to the low applied Cu^2+^ concentration. Contrary to the copper-surfactant foliar exposure results, higher Mo loading was found in wheat leaves that were exposed to Mo IONs (104.5 μg/g Mo) than those exposed to MoO_3_ NMs (72.3 μg/g Mo). The properties of the NMs (i.e., surface charge, surface coating, and hydrophobicity) and plant leaf surface characteristics (i.e., trichomes and stomata) can both influence NM-leaf surface interactions and uptake [34].

**Figure 3:**
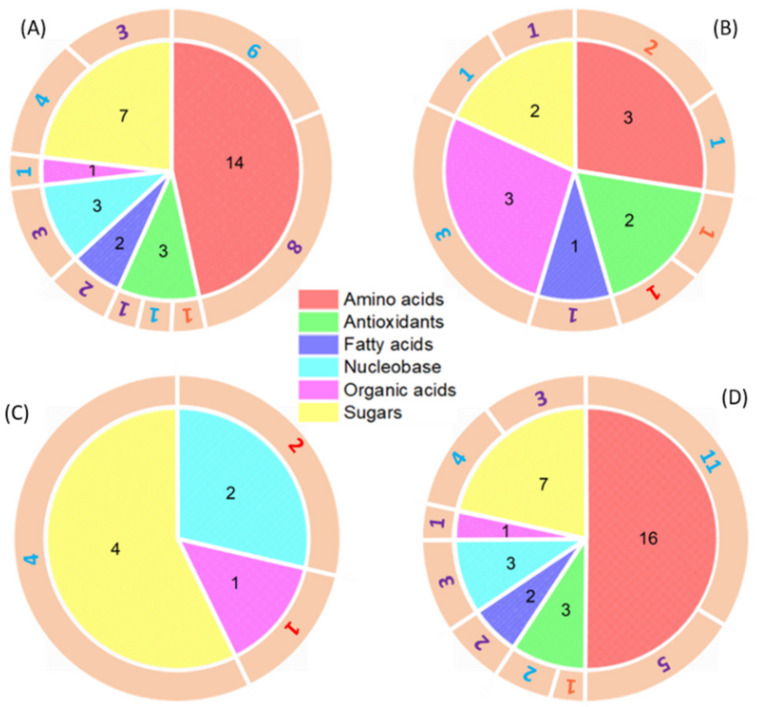
Comparison of significantly changed metabolites (/log2(fold change)/>1) in wheat leaves exposed to (**A**) Cu(OH)_2_ NMs; (**B**) MoO_3_ NMs; (**C**) Cu IONs; and (**D**) Mo IONs. Experimental conditions: SA—200 mg/L Triton^TM^ X-100 solution; Cu(OH)_2_ NMs—100 mg/L Cu(OH)_2_ NMs (as Cu content) in SA; Cu IONs—0.1 mg/L CuSO_4_·5H_2_O (as Cu content) in SA; MoO_3_ NMs—100 mg/L MoO_3_ NMs (as Mo content) in SA; and Mo IONs—35 mg/L Na_2_MoO_4_ 2H_2_O (as Mo content) in SA. The numbers in the pie chart represent the significantly changed metabolites from each metabolite category. The color of the numbers in the outer circular ring indicates fold changes of the significantly dysregulated metabolites: red (fold change > 5), orange (2 < fold change < 5), blue (0.2 < fold change < 0.5), and purple (fold change < 0.2). Three replicates were used under each condition.

The overall metabolite profiles in wheat leaves showed a clear separation between surfactant and metal-surfactant foliar exposures (Figure S6C,D). Foliar exposures to Cu(OH)_2_ NMs and Mo IONs resulted in the most significant changes of metabolite levels in wheat leaves, followed by the MoO_3_ NMs; exposure to Cu IONs had the least impact (Figure 3A–D, Table S7). These findings correspond well with the metal accumulation results on wheat leaves (Figure S6A,B), reflecting the importance of retaining metals on leaf surfaces to elicit plant’s internal response. In wheat leaves, a total of 34 dysregulated metabolites were identified for the Cu(OH)_2_ NMs and 16 for the Cu IONs foliar exposures (Table S7). When Cu(OH)_2_ NMs were applied on wheat leaves, 3 antioxidants and 14 amino acids were significantly changed, whereas no antioxidants or amino acids were significantly altered under the Cu IONs treatment (Figure 3A,C). Thus, the wheat leaf ROS defense system and changes in N metabolism were triggered by Cu(OH)_2_ NMs but not by Cu IONs. Fructose, maltose, raffinose, and sucrose from wheat leaves were significantly downregulated under both Cu(OH)_2_ NMs and Cu IONs foliar applications. The decreasing levels of these soluble sugars indicate that C fixation capacity of wheat leaves was hindered in the copper foliar exposures. Zhao et al. (2017) employed a much higher foliar dosage of ionic Cu (0.15–1.5 mg CuSO_4_/plant) versus 1.8–18 mg Cu(OH)_2_ nanopesticide/plant on lettuce, which led to contrary findings [69]. They discovered that 30 metabolites were markedly altered in response to CuSO_4_ exposure and 24 dysregulated metabolites were identified for Cu(OH)_2_ nanopesticide. Ascorbic acid, which had minor changes in the current study, decreased around 10-fold at a 1.5 mg CuSO_4_ dose and 10-fold at a 18 mg Cu(OH)_2_ nanopesticide dose [69].

Unlike the copper materials, exposure to Mo IONs resulted in 16 more dysregulated metabolites in wheat leaves than MoO_3_ NMs (Table S7). Even though a majority of the dysregulated metabolites were significantly downregulated (Figure 3B,D), aspartic acid, proline, and chlorogenic acid were significantly upregulated (fold change > 2) in wheat leaves for the MoO_3_ NMs treatment. Aspartic acid is an important factor in NH^4+^ assimilation and it can also serve as a donor for both C and N metabolism [70]. Thus, upregulation of aspartic acid may indicate the perturbation of primary N and C metabolism. Proline is a radical scavenger and can also act as an electron sink that aids in ROS defense response [71]. Dysregulation of proline has been extensively studied in plant responses to metal stressors, including metal NMs, where the proline concentration was often upregulated [39,44,45,60,72,73,74], but the level can also decrease in other cases [75]. Curcumin, as one of the antioxidants, was consistently upregulated under both MoO_3_ NMs and Mo IONs exposures. Dysregulation of antioxidant metabolites (chlorogenic acid and curcumin) suggests an induced oxidative stress response in wheat leaves to cope with increased Mo levels [75].

### 4.4. Wheat Root Responses to Metal-Surfactant Suspensions

Foliar exposure of wheat to Cu(OH)_2_ NMs and Cu IONs had little impact on Cu content in wheat roots (Figure S7A), with minimal translocation. In addition, foliar exposure to all metal-surfactant suspensions did not influence root dry biomass, nor play a major role in nutrient distribution (Figure S5B and Table S6). On the other hand, a significant amount of Mo was translocated to roots under both MoO_3_ NM and Mo ION foliar treatments (Figure S7B). The translocated fraction of MoO_3_ NMs was 65.5% (137.3 μg/g Mo in roots) and 39.0% for Mo IONs (66.9 μg/g Mo in roots). The much higher MoO_3_ NMs translocation than Cu(OH)_2_ NMs could be explained from NM properties and translocation mechanisms. NMs with a larger hydrodynamic size have shown significantly lower foliar delivery efficiency [26,30]. Since plant cell walls are mainly negatively charged, positively charged ions have shown a much higher accumulation in the apoplastic space than negative ions [76]. Compared with negatively charged MoO_3_ NMs, the movement of positively charged metal ions (e.g., Mo ions) and NMs (e.g., Cu(OH)_2_ NMs) are also likely restricted. However, the opposite result was found by Hu et al., (2020), where the positively charged NMs resulted in higher foliar transport efficiency than negative NMs in guard cells, extracellular space, and chloroplasts [26]. NM size and surface charge are not the only factors that drive NMs foliar uptake and translocation. Other parameters, such as NMs surface coating, leaf and NM hydrophilicity, solubility, binding affinity to organic phases, and plant species are likely important, but their role remains largely unknown [34].

**Figure 4:**
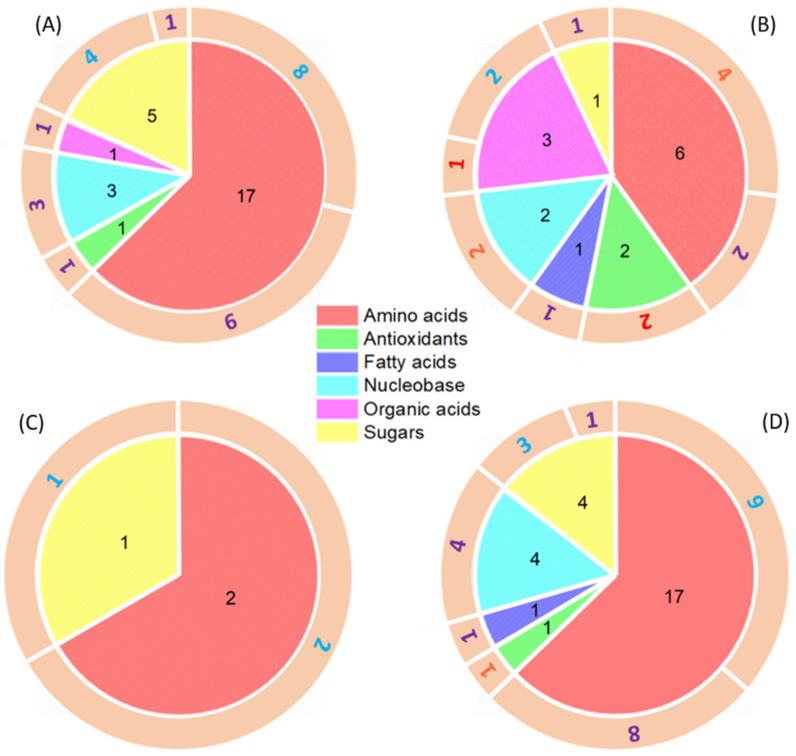
Comparison of significantly changed metabolites (/log2(fold change)/>1) in wheat roots exposed to (**A**) Cu(OH)_2_ NMs; (**B**) MoO_3_ NMs; (**C**) Cu IONs; and (**D**) Mo IONs. Experimental conditions: SA—200 mg/L Triton^TM^ X-100 solution; Cu(OH)_2_ NMs—100 mg/L Cu(OH)_2_ NMs (as Cu content) in SA; Cu IONs—0.1 mg/L CuSO_4_·5H_2_O (as Cu content) in SA; MoO_3_ NMs—100 mg/L MoO_3_ NMs (as Mo content) in SA; and Mo IONs—35 mg/L Na_2_MoO_4_·2H_2_O (as Mo content) in SA. The numbers in the pie chart represent the significantly changed metabolites from each metabolite category. The color of the numbers in the outer circular ring indicates fold changes of the significantly dysregulated metabolites: red (fold change > 5), orange (2 < fold change < 5), blue (0.2 < fold change < 0.5), and purple (fold change < 0.2). Three replicates under each condition.

The PLS-DA score plot revealed that the metabolite profiles of wheat roots exposed to metal-surfactant treatments had good separation from the surfactant-only (SA) treatment (Figure S7C,D). Physiological and nutrient distribution results showed only minor differences among different treatments (Figure S5B and Table S6). Metabolomic analysis showed that Cu(OH)_2_ NMs resulted in 27 significantly changed metabolites (/log2(fold change)/>1), while there were only 3 dysregulated metabolites discovered under Cu IONs exposure (Figure 4A,C). Except for curcumin, all the altered metabolites were downregulated (Table S8). Zhao et al., (2016) proposed that metabolites transported from leaves to roots were partially responsible for alterations of root metabolite levels [77]. In another similar study, Zhang et al. (2019) exposed spinach leaves to CeO_2_ NMs and found minimal Ce content difference in roots between the control and treatment groups [78]. Nevertheless, more profound metabolomic reprogramming was discovered in spinach roots than in leaves. However, Hong et al., (2014) treated cucumber (*Cucumis sativus*) leaves with nanoceria and found that Ce was significantly translocated to other plant sections (i.e., stems, roots, and flowers) [14]. This shows the complexity of NM foliar uptake, which often depends on NM properties, plant species, mode of application, and other environmental factors.

Even though foliar exposure to MoO_3_ NMs resulted in 110% more Mo load in roots than exposure to Mo IONs, 12 more dysregulated metabolites were identified in wheat roots under the Mo ION treatment than to MoO_3_ NMs (Figure 4B,D). Compared with leaf metabolomic results, four more significantly upregulated (fold change > 2) metabolites (lysine, phenylalanine, adenosine, and guanosine) were found in wheat roots under the MoO_3_ NMs condition. In contrast, those upregulated metabolites were all significantly downregulated (fold change < 0.2) for Mo IONs (Table S8). Amino acids (lysine and phenylalanine) play a central role in the synthesis of many cellular enzymes and in-plant detoxification towards abiotic stressors (i.e., heavy metal exposures) [79]. The significant changes of adenosine and guanosine indicated the purine metabolism pathway was disturbed. In addition, xanthine dehydrogenase/oxidase is a molybdoenzyme that has been identified in legumes. These amino acids are also involved in purine catabolism and ureide biosynthesis [63], which are essential functions for remobilizing nitrogen for plant growth and development. A recent study conducted by Huang et al. (2021) applied 200 mg/kg of MoO_3_ NMs and the corresponding Mo ionic concentrations (70 mg/kg) to wheat roots for one week [49]. Results showed that wheat leaves accumulated two times more Mo under MoO_3_ NM treatment than under Mo ionic exposure. Furthermore, nucleic acids and sugars in wheat leaves separated well from the control for MoO_3_ NMs, but only minor differences were found when wheat roots were exposed to Mo ions. The current finding demonstrates the distinct differences in NM uptake and translocation mechanisms for the two entry pathways (i.e., roots vs. foliar applications).

### 4.5. Comparison of Wheat Responses to Cu(OH)_2_ NMs and MoO_3_ NMs

The metabolic pathway analysis showed that both wheat leaves and roots were significantly perturbed for Cu(OH)_2_ NMs foliar exposure (Figure S8 and Table S9). In total, 17 pathways were perturbed in wheat leaves and 17 in roots. Glyoxylate/dicarboxylate metabolism and stilbenoid/diarylheptanoid/gingerol biosynthesis were only disturbed in wheat leaves. Chlorogenic acid is a uniquely dysregulated metabolite that is involved in the later metabolic pathway. The glyoxylate and dicarboxylate pathway is related to carbohydrate metabolism, where carbohydrates are synthesized from fatty acids [80]. On the other hand, perturbation of glutathione metabolism and valine/leucine/isoleucine biosynthesis were only found in wheat roots. These perturbed pathways are related to carbon and nitrogen metabolism and involve a uniquely altered metabolite—glutamate. Foliar exposure to Cu IONs led to 12 disturbed pathways in wheat leaves and 7 in roots (Figure S9 and Table S10). Compared with the Cu(OH)_2_ NMs foliar exposure, fewer metabolic pathways were perturbed when wheat leaves were exposed to MoO_3_ NMs (Figure S10 and Table S9). Citric acid (TCA cycle), methionine and serine (cysteine and methionine metabolism), raffinose (galactose metabolism), and serine (glycine, serine and threonine metabolism) were uniquely dysregulated metabolites in wheat leaves when they were exposed to MoO_3_ NMs. Tryptophan metabolism was the uniquely perturbed pathway in wheat roots after exposure to MoO_3_ NMs. Tryptophan is an essential component in protein synthesis and is also a central molecule that serves as a precursor for many secondary metabolites [81]. The Mo IONs treatment resulted in mostly downregulated metabolites and very similar perturbation pathways between wheat leaves and roots (Figure S11 and Table S10). Valine, isoleucine, and leucine were significantly downregulated under the Mo IONs treatment that led to the perturbation of valine/leucine/isoleucine biosynthesis.

**Figure 5:**
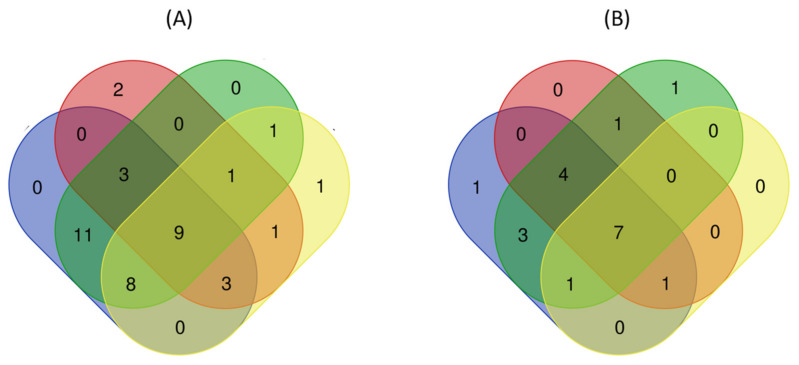
Venn diagram of (**A**) dysregulated metabolites and (**B**) perturbed metabolic pathways in wheat after one-week foliar exposure to NMs suspensions. Legend: 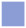 wheat leaves exposed to Cu(OH)_2_ NMs; 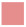 wheat leaves exposed to MoO_3_ NMs; 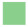 wheat roots exposed to Cu(OH)_2_ NMs; 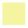 wheat roots exposed to MoO_3_ NMs.

In general, the Venn diagram demonstrates that exposure to Cu(OH)_2_ NMs reprogramed the wheat metabolic profile more than exposure to MoO_3_ NMs (Figure 5). This finding was quite interesting, considering only a very small amount of Cu was dissolved from Cu(OH)_2_ NMs and even less was translocated to wheat roots. On the other hand, a much higher amount of Mo was dissolved from MoO_3_ NMs and 65.5% of Mo was translocated to wheat roots (Figure S7A,B). Compared with MoO_3_ NMs exposure, there were 10 more dysregulated metabolites found in wheat leaves and 9 more discovered in roots when Cu(OH)_2_ NMs applied (Table S11). Even though four dysregulated metabolites (i.e., ribitol, citric acid, adenine, and ascorbic acid) only responded to MoO_3_ NMs, there were no uniquely perturbed pathways found when compared to Cu(OH)_2_ NMs (Table S12). Conversely, glyoxylate/dicarboxylate metabolism from wheat leaves and valine/leucine/isoleucine biosynthesis from wheat roots uniquely responded to Cu(OH)_2_ NMs.

## 5. Conclusions

When Triton^TM^ X-100 surfactant (SA) was applied to wheat leaves, minimal changes were observed in physiological responses and nutrient distributions. However, metabolite profiles were significantly reprogrammed. For instance, leucine, lysine, phenylalanine, proline, serine, tryptophan, linolenic acid, adenosine, guanosine, uridine, and trehalose were significantly changed (with fold change > 5) in wheat leaves and roots. In addition, 17 metabolic pathways were perturbed in wheat leaves and 15 in roots.

Adding the surfactant significantly promoted applied NMs and ionic metal deposition to wheat leaves. A significant amount of Cu (649.8 μg/g) was detected in wheat leaves under Cu(OH)_2_ NMs, which resulted in 34 dysregulated metabolites. Since only 0.1% of Cu(OH)_2_ NMs dissolved in SA, Cu IONs had minimum effect on Cu accumulation or metabolic alterations. Even though minimum translocation was observed for Cu(OH)_2_ NMs, there were 27 significantly altered metabolites discovered in wheat roots. In contrast, a large percentage of the MoO_3_ NMs dissolved in SA (e.g., 35.8%), which led to 32.2 μg/g more Mo and 16 more dysregulated metabolites detected in wheat leaves under MoO_3_ IONs treatment. Even MoO_3_ NMs resulted in 110% more Mo loading in wheat roots than Mo IONs, 16 additional dysregulated metabolites were discovered for the Mo IONs treatment.

Due to differences in NM properties (e.g., hydrodynamic diameter, zeta potential, and dissolution extent), metabolite pathway analysis showed Cu(OH)_2_ NMs reprogramed wheat metabolites profile more than MoO_3_ NMs. Even though ribitol, citric acid, adenine, and ascorbic acid only responded to MoO_3_ NMs, no uniquely disturbed pathways were discovered. Conversely, Cu(OH)_2_ NMs resulted in uniquely disturbed glyoxylate/dicarboxylate metabolism in wheat leaves and the perturbation valine/leucine/isoleucine metabolism in wheat roots.

Overall, this study revealed that foliar application of NM-surfactant suspensions could deposit more NMs on the leaves and significantly reprogram the metabolic profile of early-stage wheat seedlings. A thorough understanding of the plant’s internal status changes could benefit surfactant innovation, thus promoting more sustainable and efficient NMs foliar application in agriculture. In future studies, other omics techniques, such as proteomics, transcriptomics, and genomics can help to illustrate plant biochemical responses to NMs exposures. Here we observed plant responses after one-week foliar exposure, as a proof-of-concept; future studies should consider plant responses with longer time durations. Furthermore, other agricultural-related surfactants and environmental conditions could be considered to better mimic field applications.

## Data Availability

All data that supported findings from the current study is available upon request.

## Supplementary Materials

The following are available online at https://www.mdpi.com/article/10.3390/nano11113073/s1, Figure S1: Transmission electron microscope (TEM) imaging of (**A**) Cu(OH)_2_ NMs and (**B**) MoO_3_ NMs; Figure S2: Water droplet (5 μl) dispersion test of the (**a**) 100, (**b**) 200, (**c**) 250, (**d**) 500, and (**e**) 1000 mg/L Triton^TM^ X-100 solutions on the 3-weeks old wheat leaf; Figure S3: Comparison of Mo content in wheat leaves after one-week foliar exposure to deionized water (DI) or 100 mg/L MoO_3_ NMs suspensions in DI; Figure S4: Comparison of dry biomass data after one-week foliar exposure to deionized water (DI) or surfactant solutions (SA; Figure S5: Dry biomass of wheat (**A**) leaves and (**B**) roots after one-week foliar exposure to metal-surfactant suspensions; Figure S6: Metal accumulation and metabolite alterations in wheat leaves after one-week foliar exposure to NM-surfactant or ion-surfactant: (**A**) Cu and (**B**) Mo level in wheat leaves; PLS-DA score plot of the overall metabolite profile due to (**C**) Cu(OH)_2_ NMs and Cu IONs; and (**D**) MoO_3_ NMs and Mo IONs foliar exposures; Figure S7: Metal accumulation and metabolite alterations in wheat roots after one-week foliar exposure to NM-surfactant or ion-surfactant: (**A**) Cu and (**B**) Mo level in wheat leaves; PLS-DA score plot of the overall metabolite profile due to (**C**) Cu(OH)_2_ NMs and Cu IONs; and (**D**) MoO_3_ NMs and Mo IONs foliar exposures; Figure S8: Significant changes in metabolite pathway of wheat after one-week foliar exposure to 100 mg/L of Cu(OH)_2_ NMs suspension; Figure S9: Significant changes in metabolite pathway of wheat after one-week foliar exposure to 100 mg/L of MoO_3_ NMs suspension; Figure S10: Significant changes in metabolic pathways of wheat after one-week foliar exposure to 0.1 mg/L of CuSO_4_ · 5H_2_O solution. The color scale indicates the fold changes compared to DI only; Figure S11: Significant changes in metabolic pathways of wheat after one-week foliar exposure to 35 mg/L of Na_2_MoO_4_ · 2H_2_O solution; Table S1: List of metabolites parameters measured by LC/MS; Table S2: Hydrodynamic diameter and zeta potential of Cu(OH)_2_ and MoO_3_ NMs at time 0; Table S3: Nutrient distributions in wheat leaves and roots after one-week foliar exposure to deionized water (DI) or surfactant solutions (SA); Table S4: Significantly altered metabolites in wheat leaves and roots after one-week foliar exposure to surfactant solutions; Table S5: Pathway analysis results in wheat plant tissues after one-week foliar exposures to 200 mg/L of surfactant solutions; Table S6: Nutrient distributions in wheat leaves and roots after one-week foliar exposure to metal-surfactant suspensions; Table S7: Significantly altered metabolites in wheat leaves after one-week foliar exposure to metal-surfactant suspensions; Table S8: Significantly altered metabolites in wheat roots after one-week foliar exposure to metal-surfactant suspensions; Table S9: Perturbed pathway analysis in wheat plant tissues after one-week foliar exposures to 100 mg/L of Cu (OH)_2_ and MoO_3_ NMs suspensions; Table S10: Perturbation pathway analysis in wheat plant tissues after one-week foliar exposures to 0.1 mg/L of CuSO_4_ · 5H_2_O _4_ and 35 mg/L of Na_2_MoO_4_ · 2H_2_O solution; Table S11: Venn diagram results of dysregulated metabolites in wheat after one-week foliar exposure to 100 mg/L of Cu (OH)_2_ and MoO_3_ NMs suspensions; Table S12: Venn diagram results of perturbed metabolic pathways in wheat after one-week foliar exposure to 100 mg/L of Cu (OH)_2_ and MoO_3_ NMs suspensions.
