# Supplementary material for: Metabolomic Response of Early-Stage Wheat (Triticum aestivum) to Surfactant-Aided Foliar Application of Copper Hydroxide and Molybdenum Trioxide Nanoparticles
Source: Nanomaterials (Basel). 2021 Nov 15;11(11):3073. doi: 10.3390/nano11113073 (PMC8622224; doi:10.3390/nano11113073)
Supplement: Supplementary file 1 [file nanomaterials-11-03073-s001.zip › nanomaterials-1413612-supplementary.pdf]

nanomaterials

Supporting Information

Metabolomic Response of Early-Stage Wheat (*Triticum aestivum*) to Surfactant-Aided Foliar Application of Copper Hydroxide and Molybdenum Trioxide Nanoparticles

Xiangning Huang [1] and Arturo A. Keller [1,2,*]

[1] Center for Environmental Implications of Nanotechnology, University of California, Santa Barbara, CA 93106, USA; xiangningh@ucsb.edu
[2] Bren School of Environmental Science and Management, University of California, Santa Barbara, CA 93106, USA
* Correspondence: keller@bren.ucsb.edu

Citation: Huang, X.; Keller, A.A. Metabolomic Response of Early-Stage Wheat (*Triticum aestivum*) to Surfactant-Aided Foliar Application of Copper Hydroxide and Molybdenum Trioxide Nanoparticles. *Nanomaterials* **2021**, *11*, 3073. https://doi.org/10.3390/nano11113073

Academic Editor: Linda J. Johnston

Received: 23 September 2021
Accepted: 6 November 2021
Published: 15 November 2021

Publisher's Note: MDPI stays neutral with regard to jurisdictional claims in published maps and institutional affiliations.

Copyright: © 2021 by the authors. Licensee MDPI, Basel, Switzerland. This article is an open access article distributed under the terms and conditions of the Creative Commons Attribution (CC BY) license (http://creativecommons.org/licenses/by/4.0/).

LC/MS/MS sample preparation and measurements:

Vial 1: antioxidants were separated using an Agilent ZORBAX StableBond 80 AC18 4.6 mm × 50 mm, 3.5 µm column with mobile phase A as 0.1% formic acid with 5 mM ammonium formate in water and mobile phase B as methanol.

Vial 2: for quantifying organic acids/phenolics and nucleobase/side/tides, 40 µl of 0.2 mg/ml citric acid-D4 and 40 µl of 0.25 mg/ml thymine-13C1 and 120 µl water were added. The Agilent Polaris 3 C-18 Ether 150 × 3.0 mm column was used and the mobile phases were: (A) 0.1% formic acid in water and (B) 0.1% formic acid in methanol.

Vial 3: for the quantification of amino acids and sugar/sugar alcohols, 30 µl of 10 µg/ml alaine-D3; D2 glycine; D3 aspartic acid; D8 lysine; glutamic acid-15N1; methionine-D8 and 50 µl of 0.1 mg/ml glucose-D2 were added. Then the sample was dried with nitrogen and reconstituted to 80% acetonitrile in water for the LC/MS analysis. The Agilent InfinityLab Poroshell 120 HILIC-Z 2.1 × 100 mm, 2.7 µm column was used and the mobile phases for amino acids were: (A) 10% (200 mM ammonium formate in water at pH 3) in water/ (B) 10% (200 mM ammonium formate in water at pH 3) in acetonitrile. The mobile phases for sugar/sugar alcohols were A) 0.3% ammonium hydroxide in water/ (B) 0.3% ammonium hydroxide in acetonitrile.

Vial 4: for the fatty acids measurement, after adding 50 µl/ml of 0.2 mg of each arachidic acid-D39 and myristic acid-D3, vial 4 was reconstituted in a solution containing acetonitrile: isopropanol:water = 65:30:5. The column used here was the same as vial 2, mobile phase A and B were 10 mM ammonia formate in 40% acetonitrile in water (with 0.1% formic acid) and 10 mM ammonia formate in 90% isopropanol in water (with 0.1% formic acids), respectively.

Table S1. List of metabolites parameters measured by LC/MS.

Metabolomic Compound	RT (min)	Precursor Ion (m/z)	Product Ion (m/z)	Linearity (R^2)
		Amino acids		
Phenylalanine	2.95	166.1	120.1	0.9933
Leucine	3.38	132.1	86.1	0.9987
Tryptophan	3.41	205.1	188.0	0.9945
Isoleucine	3.75	132.1	86.1	0.9966
Methionine	4.22	150.1	104.0	0.9997
Valine	4.95	118.1	72.1	0.9964
Proline	4.96	116.1	70.1	0.9989
Tyrosine	5.01	182.1	136.1	0.9933
Cysteine	5.63	122.0	59.1	0.9882
Alanine	6.61	90.1	44.2	0.9967
Threonine	6.72	120.1	74.1	0.9974
Homoserine	6.91	120.1	74.1	0.9988
Glycine	7.00	76.0	30.3	0.9986
Glutamine	7.23	147.1	84.1	0.9973
Serine	7.26	106.1	88.1	0.9969
Asparagine	7.31	133.1	87.1	0.9988
Glutamic acid	7.68	148.1	84.1	0.9976
Citrulline	7.89	176.1	159.1	0.9896
Aspartic acid	8.38	134.0	88.1	0.9894
Histidine	9.06	156.1	110.1	0.9948
Arginine	9.54	175.1	70.1	0.9990
Lysine	10.16	147.1	84.1	0.9678
Ornithine	10.28	133.1	116.0	0.9752
		Antioxidants		
Glutathione reduced	1.22	308.1	179.0	0.9986
Chlorogenic acid	6.19	353.1	191.1	0.9993
Curcumin	6.33	367.1	217.1	0.9518
Vanillic acid	6.60	167.0	152.1	0.9963
2-hydroxycinnamic acid	7.37	163.0	119.1	0.9972
L-Dehydroascorbic acid	8.00	173.0	158.1	0.9752
4-(Trifuoromethyl)cinnamic acid	8.26	215.0	171.1	0.9968
a-Tocopherol	11.00	431.4	165.1	0.9716
		Fatty acids		
Linolenic acid	4.33	323.2	227.1	0.9759
Myristic acid	4.64	273.2	227.2	0.9843
Linoleic acid	4.91	325.2	279.1	0.9868
Pentadecanoic acid	5.17	287.2	241.2	0.9870
Palmitic acid	5.70	301.2	255.2	0.9943
Heptadecanoic acid	6.14	315.3	269.2	0.9760
Stearic acid	6.49	329.3	283.2	0.9981
Arachidic acid	7.05	357.3	311.3	0.9894
		Nucleobase/side/tide		
Cytosine	1.94	112.1	95.0	0.9816
CMP	2.76	324.1	112.0	0.9909
Cytidine	2.90	244.1	112.0	0.9942
Adenine	3.08	136.1	119.0	0.9819
Guanine	3.34	152.1	135.0	0.9930
Uracil	3.52	113.0	70.0	0.9781

AMP	4.84	348.1	136.0	0.9942
Hypoxanthine	5.28	137.0	110.0	0.9917
Uridine	6.33	245.1	113.0	0.9969
Xanthine	6.40	153.0	110.0	0.9971
Adenosine	6.67	268.1	136.0	0.9965
Thymine	6.71	127.1	110.0	0.9861
Guanosine	6.91	284.1	152.0	0.9999
Inosine	6.91	269.1	137.0	0.9851
Thymidine	7.28	243.1	127.0	0.9984
Organic acids/Phenolics				
Glycolic acid	2.04	75.0	47.0	0.9953
Malic acid	2.07	133.0	114.9	0.9984
Citric acid	2.17	191.0	110.8	0.9968
Lactic acid	2.23	89.1	43.1	0.9956
Succinic acid	2.31	117.0	72.9	0.9981
Pyruvic acid	2.36	87.0	43.1	0.9937
Gallic acid	2.49	169.0	125.1	0.9936
Glutaric acid	2.62	131.0	86.9	0.9952
Fumaric acid	2.67	115.0	70.9	0.9942
Ascorbic acid	2.67	175.0	114.9	0.9950
Caffeic acid	4.58	179.0	135.1	0.9916
p-coumaric acid	4.87	163.0	119.1	0.9911
Ferulic acid	5.09	193.1	134.1	0.9981
Benzoic acid	5.21	121.0	77.1	0.9928
Salicylic acid	5.96	137.0	93.0	0.9931
Sugar/Sugar alcohols				
Ribose	1.18	149.0	89.0	0.9933
L-fucose	1.35	163.1	89.0	0.9871
Xylose/Arabinose	1.43	149.0	89.0	0.9847
Ribitol/Xylitol	1.61	151.1	89.0	0.9973
Fructose	1.72	179.1	89.0	0.9962
Mannose	1.93	179.1	89.0	0.9974
Glucose/Galactose	2.19	179.1	89.0	0.9986
Sucrose	3.81	341.1	179.0	0.9963
Maltose	4.26	341.1	161.1	0.9962
Lactose	4.57	341.1	161.1	0.9976
Trehalose	4.79	341.1	179.0	0.9962
Raffinose	6.03	503.2	179.0	0.9943
Galactinol	6.17	341.1	179.0	0.9926

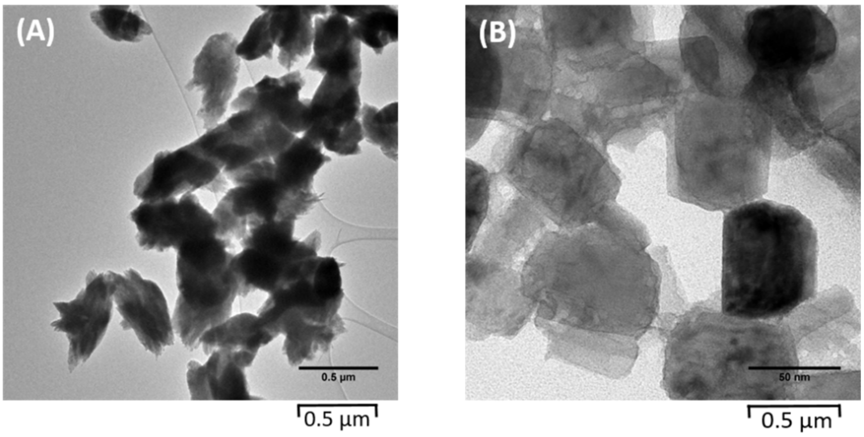

Figure S1. Transmission electron microscope (TEM) imaging of (**A**) $Cu(OH)_2$ NMs and (**B**) MoO_3 NMs.

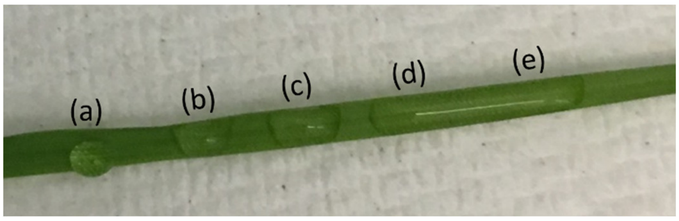

Figure S2. Water droplet (5 µl) dispersion test of the (**a**) 100, (**b**) 200, (**c**) 250, (**d**) 500, and (**e**) 1000 mg/L Triton™ X-100 solutions on the 3-weeks old wheat leaf. When the surfactant concentration was above 200 mg/L, the droplets began to roll and drip away.

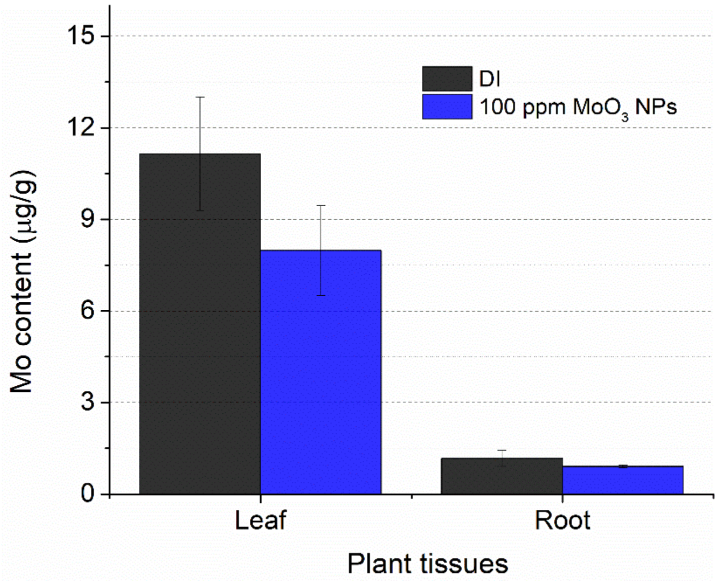

Figure S3. Comparison of Mo content in wheat leaves after one-week foliar exposure to deionized water (DI) or 100 mg/L MoO_3 NMs suspensions in DI, respectively. Four replicates were utilized under each condition.

Table S2. Hydrodynamic diameter and zeta potential of $Cu(OH)_2$ and MoO_3 NMs at time 0.

Conditions	Hydrodynamic diameter mV	Zeta potential ζ
$Cu(OH)_2$ nanoparticle		
Deionized water only	944.2 ± 13.7	4.84 ± 0.92
200 mg/L Triton X-100	1074 ± 168.7	15.9 ± 0.93[A]
MoO3 nanoparticle		
Deionized water only	299.9 ± 18.2	−65.0 ± 1.03
200 mg/L Triton X-100	301.3 ± 8.1	−51.9 ± 0.79[A]

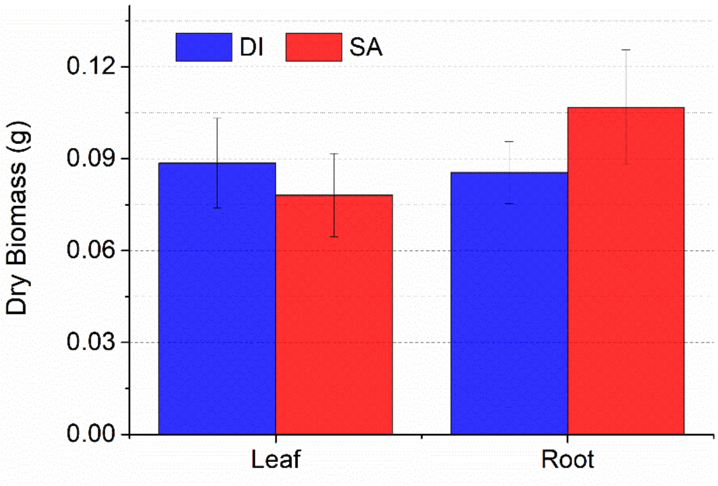

Figure S4. Comparison of dry biomass data after one-week foliar exposure to deionized water (DI) or surfactant solutions (SA), respectively. Six replicates were utilized under each condition.

Table S3. Nutrient distributions in wheat leaves and roots after one-week foliar exposure to deionized water (DI) or surfactant solutions (SA).

Conditions	Macronutrients (mg/g)				Micronutrients (μg/g)				
Wheat-leaves	Mg	P	K	Ca	Mn	Fe	Cu	Zn	Mo
DI	5.89	5.32	81.47	2.63	308.78	63.93	56.96	51.81	21.12
SA	3.79	4.31	55.98	2.38	210.15	54.50	60.18	52.93	8.63 [A]
Wheat-roots									
DI	1.64	1.29	22.26	1.11	341.42	233.93	207.80	83.91	2.18
SA	2.63	1.79	32.21	1.16	284.06	207.68	193.56	66.90	1.22

Note: The upper letter represented the significant difference ($p < 0.05$) compared with the DI group.

Table S4. Significantly altered metabolites in wheat leaves and roots after one-week foliar exposure to surfactant solutions.

leaf	FC	root	FC
	Amino acids		
Alanine	5.93	Alanine	4.33
Arginine	4.94	Arginine	2.97
Asparagine	3.04	Asparagine	5.45
Aspartic acid	2.60	Aspartic acid	1.95
Glutamine	3.56	Glutamine	3.74
Homoserine	3.76	Homoserine	3.50
Isoleucine	5.42	Isoleucine	3.42
Leucine	13.43	Leucine	8.10
Lysine	10.44	Lysine	6.64
Methionine	3.96	Methionine	3.05
Phenylalanine	11.96	Phenylalanine	11.83
Proline	7.58	Proline	5.12
Serine	>5	Serine	6.81
Threonine	4.04	Threonine	3.04
Tryptophan	8.86	Tryptophan	9.37
Tyrosine	3.83	Tyrosine	3.14
Valine	4.58	Valine	2.89
Ascorbic acid	>5	Citrulline	2.64
Glutamic acid	1.35		
Histidine	1.95		
	Antioxidants		
Curcumin	0.30	Curcumin	0.41
Glutathione reduced	2.61	Glutathione reduced	>5
Chlorogenic acid	2.27		
	Fatty acids		
Linoleic acid	1.64	Linoleic acid	1.78
Linolenic acid	>5	Linolenic acid	>5
	Nucleobase/side/tide		
Adenosine	>5	Adenosine	>5
Guanosine	>5	Guanosine	>5
Uridine	>5	Uridine	>5
		Cytidine	>5
	Organic acids/phenolics		
Citric acid	0.70	Citric acid	0.69
Succinic acid (N)	4.97	Pyruvic acid	0.47
	Sugar/alcohols		
Fructose	4.09	Fructose	3.06
Glucose/Galactose	4.09	Glucose/Galactose	2.98
Mannose	3.84	Mannose	3.24
Raffinose	4.40	Raffinose	1.89
Sucrose	2.72	Sucrose	0.70
Trehalose	>5	Trehalose	>5
Maltose	2.81		

Table S5. Pathway analysis results in wheat plant tissues after one-week foliar exposures to 200 mg/L of surfactant solutions.

Disturbed pathways	*p* value	Impact factor
Leaf-SA		
Phenylalanine metabolism	9.409×10^{-6}	0.42
Galactose metabolism	1.701×10^{-5}	0.13
Starch and sucrose metabolism	2.848×10^{-5}	0.50
Aminoacyl-tRNA biosynthesis	4.071×10^{-5}	0.11
Tryptophan metabolism	4.819×10^{-5}	0.17
Alanine, aspartate and glutamate metabolism	5.967×10^{-5}	0.64
alpha-Linolenic acid metabolism	1.186×10^{-4}	0.11
Glycine, serine and threonine metabolism	1.664×10^{-4}	0.37
Cysteine and methionine metabolism	1.983×10^{-4}	0.13
Tyrosine metabolism	1.089×10^{-3}	0.17
Isoquinoline alkaloid biosynthesis	1.089×10^{-3}	0.41
Arginine biosynthesis	1.649×10^{-3}	0.40
Arginine and proline metabolism	2.596×10^{-3}	0.38
Citrate cycle (TCA cycle)	4.026×10^{-3}	0.16
Linoleic acid metabolism	5.253×10^{-3}	1.00
Glutathione metabolism	1.474×10^{-2}	0.40
Stilbenoid, diarylheptanoid and gingerol biosynthesis	3.016×10^{-2}	0.11
Root-SA		
Starch and sucrose metabolism	2.750×10^{-5}	0.50
Phenylalanine metabolism	1.185×10^{-4}	0.42
alpha-Linolenic acid metabolism	3.050×10^{-4}	0.11
Galactose metabolism	7.278×10^{-4}	0.13
Tryptophan metabolism	7.542×10^{-4}	0.17
Aminoacyl-tRNA biosynthesis	1.585×10^{-3}	0.11
Valine, leucine and isoleucine biosynthesis	2.190×10^{-3}	0.11
Alanine, aspartate and glutamate metabolism	2.533×10^{-3}	0.64
Isoquinoline alkaloid biosynthesis	2.838×10^{-3}	0.41
Glycine, serine and threonine metabolism	4.384×10^{-3}	0.37
Cysteine and methionine metabolism	4.719×10^{-3}	0.14
Tyrosine metabolism	1.108×10^{-2}	0.17
Citrate cycle (TCA cycle)	1.220×10^{-2}	0.16
Linoleic acid metabolism	1.618×10^{-2}	1.00
Glutathione metabolism	4.485×10^{-2}	0.40

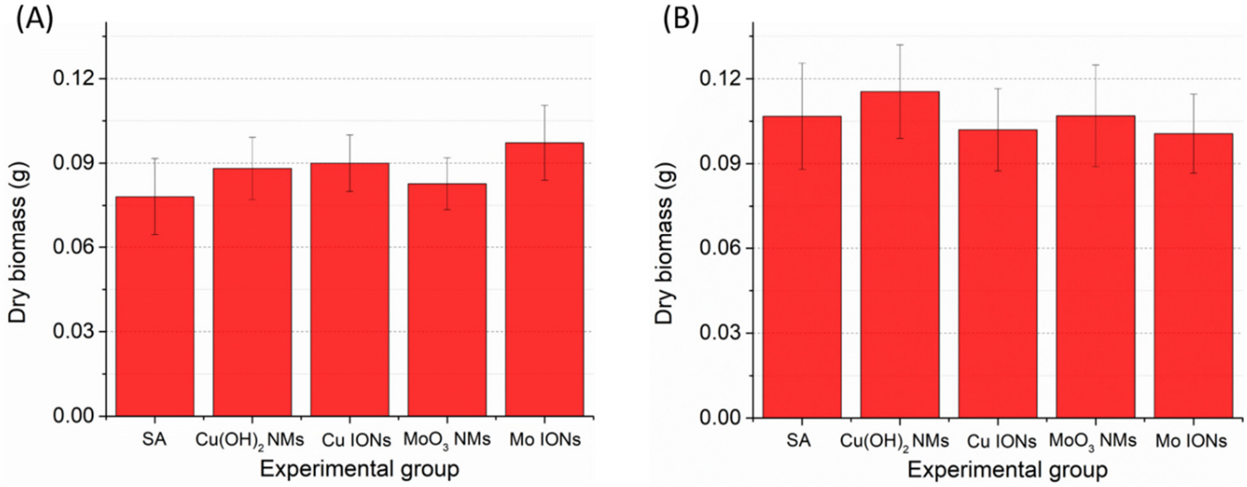

Figure S5. Dry biomass of wheat (**A**) leaves and (**B**) roots after one-week foliar exposure to metal-surfactant suspensions. Six replicates were utilized under each condition. Experimental conditions: SA—200 mg/L Triton™ X-100 solution; $Cu(OH)_2$ NMs—100 mg/L $Cu(OH)_2$ NMs (as Cu content) in SA; Cu IONs—0.1 mg/L $CuSO_4 \cdot 5H_2O$ (as Cu content) in SA; MoO_3 NMs—100 mg/L MoO_3 NMs (as Mo content) in SA; and Mo IONs—35 mg/L $Na_2MoO_4 \cdot 2H_2O$ (as Mo content) in SA.

Table S6. Nutrient distributions in wheat leaves and roots after one-week foliar exposure to metal-surfactant suspensions.

Conditions	Macronutrients (mg/g)				Micronutrients (μg/g)				
Wheat-leaves	Mg	P	K	Ca	Mn	Fe	Cu	Zn	Mo
SA	3.79	4.31	55.98	2.38	210.15	54.50	60.18	52.93	8.63
a	4.36	4.57	60.54	3.92	303.00	60.56	649.84^{A}	81.39^{A}	19.43
b	5.14	4.92	65.21	2.98	318.02	69.54	66.24	51.44	18.07
c	3.86	4.56	53.45	1.32	232.13	55.35	45.28	43.01	72.29^{A}
d	4.85	5.09	67.35	2.56	281.00	61.47	63.85	52.22	104.52^{B}
Wheat-roots									
SA	2.63	1.79	32.21	1.16	284.06	207.68	193.56	66.90	1.22
a	2.29	2.27	32.60	0.65	348.81	127.87	180.33	42.35	2.07
b	2.77	2.32	29.35	1.07	657.26^{A}	234.61	192.52	64.93	2.12
c	3.02	1.93	45.65	0.74	461.21	248.44	168.90	46.64	137.31^{B}
d	3.43	1.05	36.31	0.87	295.91	257.89	123.32	45.69	66.90^{A}

Note: The upper letter represented the significant difference ($p < 0.05$) compared with the SA group. Experimental conditions: SA—200 mg/L Triton™ X-100 solution; $Cu(OH)_2$ NMs—100 mg/L $Cu(OH)_2$ NMs (as Cu content) in SA; Cu IONs—0.1 mg/L $CuSO_4 \cdot 5H_2O$ (as Cu content) in SA; MoO_3 NMs—100 mg/L MoO_3 NMs (as Mo content) in SA; and Mo IONs—35 mg/L $Na_2MoO_4 \cdot 2H_2O$ (as Mo content) in SA.

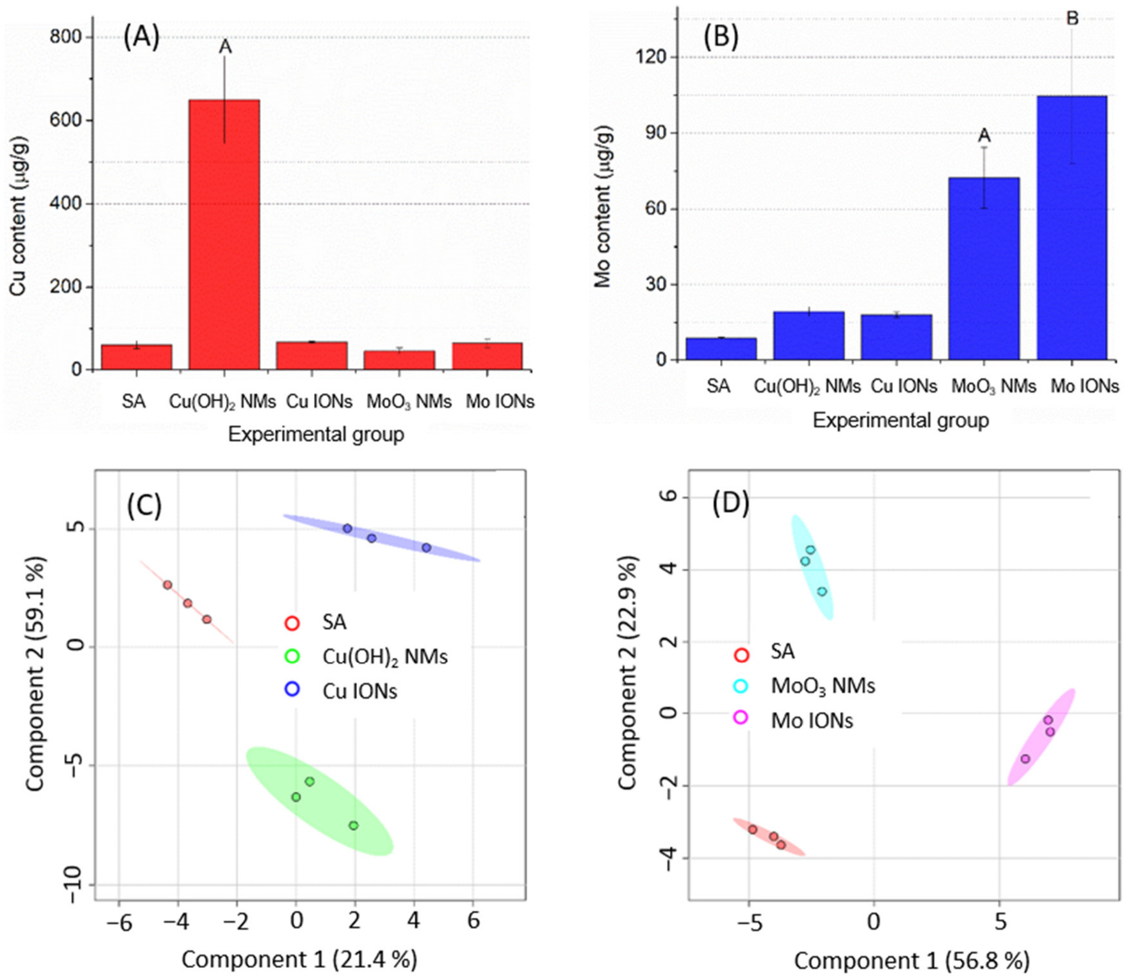

Figure S6. Metal accumulation and metabolite alterations in wheat leaves after one-week foliar exposure to NM-surfactant or ion-surfactant: (**A**) Cu and (**B**) Mo level in wheat leaves; PLS-DA score plot of the overall metabolite profile due to (**C**) $Cu(OH)_2$ NMs and Cu IONs; and (**D**) MoO_3 NMs and Mo IONs foliar exposures; Experimental conditions: SA—200 mg/L Triton™ X-100 solution; $Cu(OH)_2$ NMs—100 mg/L $Cu(OH)_2$ NMs (as Cu content) in SA; Cu IONs—0.1 mg/L $CuSO_4 \cdot 5H_2O$ (as Cu content) in SA; MoO_3 NMs—100 mg/L MoO_3 NMs (as Mo content) in SA; and Mo IONs—35 mg/L $Na_2MoO_4 \cdot 2H_2O$ (as Mo content) in SA.

Table S7. Significantly altered metabolites in wheat leaves after one-week foliar exposure to metal-surfactant suspensions.

$Cu(OH)_2$ NMs	FC	Cu IONs	FC	MoO_3 NMs	FC	Mo IONs	FC
				Amino acids			
Methionine	0.22	Methionine	0.73	Asparagine	1.49	Asparagine	0.62
Serine	<0.2	Serine	1.06	Aspartic acid	2.98	Aspartic acid	0.64
Tryptophan	0.09	Tryptophan	1.45	Glutamine	0.31	Glutamine	0.46
Tyrosine	0.20	Tyrosine	0.72	Methionine	0.62	Methionine	0.24
Alanine	0.22	Glutamic acid	1.18	Phenylalanine	1.49	Phenylalanine	0.09
Arginine	0.20			Proline	2.47	Proline	0.18
Glutamine	0.56			Serine	1.46	Serine	<0.2
Histidine	0.53					Alanine	0.24
Homoserine	0.32					Arginine	0.22
Isoleucine	0.15					Histidine	0.52
Leucine	0.09					Homoserine	0.33
Lysine	0.14					Isoleucine	0.21
Proline	0.15					Leucine	0.12
Phenylalanine	0.07					Lysine	0.08
Threonine	0.30					Threonine	0.32
Valine	0.27					Tryptophan	0.33
						Tyrosine	0.25
						Valine	0.30
				Antioxidants			
Chlorogenic acid	0.36			Chlorogenic acid	3.64	Chlorogenic acid	0.40
Curcumin	2.25			Curcumin	14.27	Curcumin	2.57
Glutathione reduced	<0.2					Glutathione reduced	0.22
				Fatty acids			
Linoleic acid	0.18			Linoleic acid	<0.2	Linoleic acid	<0.2
Linolenic acid	<0.2					Linolenic acid	<0.2
				Nucleobase/side/tide			
Uridine	<0.2	Uridine	1.24	Adenine	>5	Adenosine	<0.2
Adenosine	<0.2	Adenine	>5			Guanosine	<0.2
Guanosine	<0.2	Cytidine	>5			Uridine	<0.2
				Organic acids/phenolics			
Aspartic acid	0.58	Aspartic acid	1.59	Malic acid	0.26	Malic acid	0.69
Malic acid	0.79	Pyruvic acid	>5	Citric acid	0.49	Ascorbic acid	0.19
Succinic acid	0.20			Succinic acid	0.23		
				Sugar/alcohols			
Fructose	0.14	Fructose	0.43	Fructose	0.52	Fructose	0.16
Maltose	0.27	Maltose	0.27	Raffinose	0.29	Raffinose	0.07
Mannose	0.30	Mannose	1.00	Sucrose	0.77	Sucrose	0.28
Raffinose	0.11	Raffinose	0.26	Trehalose	<0.2	Trehalose	<0.2
Sucrose	0.28	Sucrose	0.27	Ribitol/Xylitol	1.15	Glucose/Galactose	0.22
Trehalose	<0.2	Trehalose	0.57			Maltose	0.26
Glucose/Galactose	0.28					Mannose	0.22

Experimental conditions: Cu(OH)2 NMs—100 mg/L Cu(OH)2 NMs (as Cu content) in SA; Cu IONs—0.1 mg/L CuSO4 · 5H2O (as Cu content) in SA; MoO3 NMs—100 mg/L MoO3 NMs (as Mo content) in SA; and Mo IONs—35 mg/L Na2MoO4 · 2H2O (as Mo content) in SA.

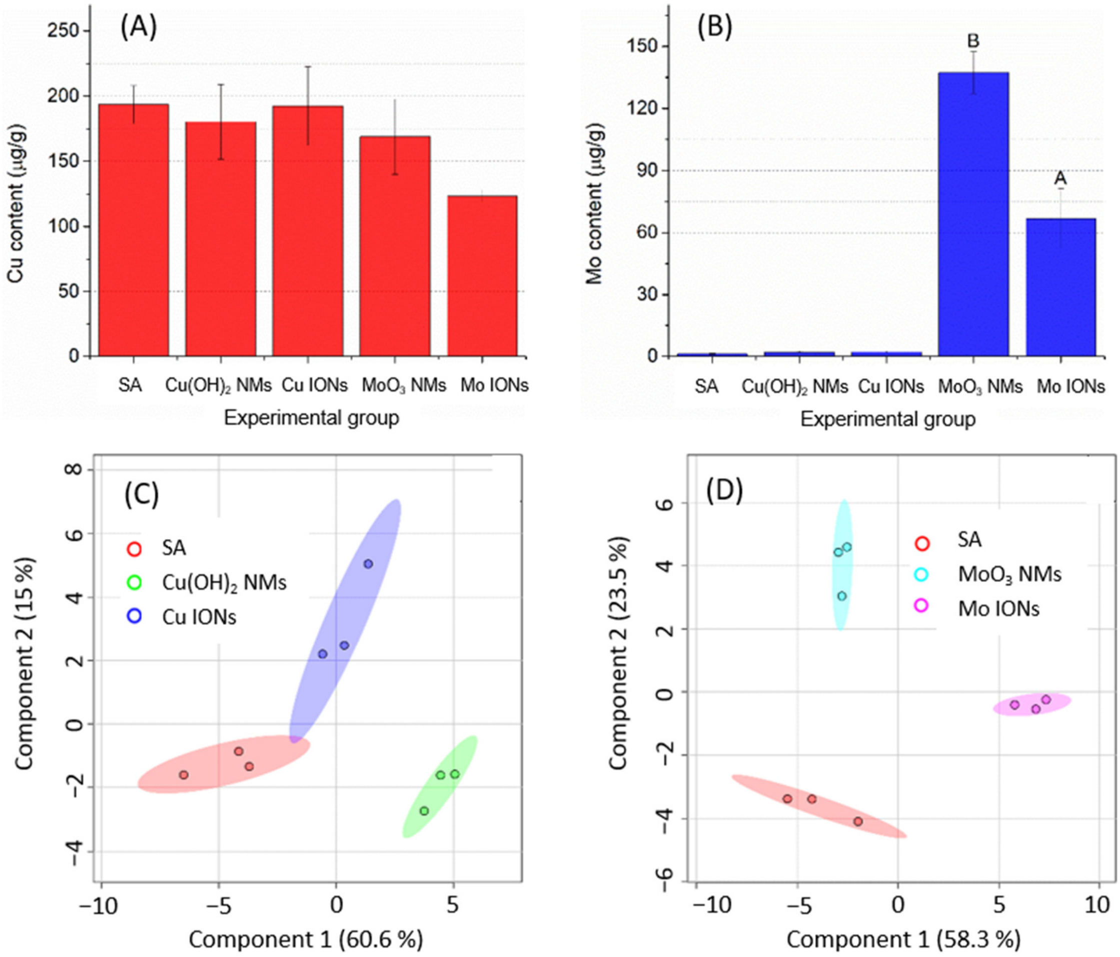

Figure S7. Metal accumulation and metabolite alterations in wheat roots after one-week foliar exposure to NM-surfactant or ion-surfactant: (**A**) Cu and (**B**) Mo level in wheat leaves; PLS-DA score plot of the overall metabolite profile due to (**C**) $Cu(OH)_2$ NMs and Cu IONs; and (**D**) MoO_3 NMs and Mo IONs foliar exposures Experimental conditions: SA—200 mg/L Triton™ X-100 solution; $Cu(OH)_2$ NMs—100 mg/L $Cu(OH)_2$ NMs (as Cu content) in SA; Cu IONs—0.1 mg/L $CuSO_4 \cdot 5H_2O$ (as Cu content) in SA; MoO_3 NMs—100 mg/L MoO_3 NMs (as Mo content) in SA; and Mo IONs—35 mg/L $Na_2MoO_4 \cdot 2H_2O$ (as Mo content) in SA.

Table S8. Significantly altered metabolites in wheat roots after one-week foliar exposure to metal-surfactant suspensions.

$Cu(OH)_2$ NMs	FC	Cu IONs	FC	MoO_3 NMs	FC	Mo IONs	FC
				Amino acids			
Alanine	0.21	Alanine	0.65	Asparagine	0.16	Asparagine	0.12
Asparagine	0.09	Asparagine	0.51	Aspartic acid	1.37	Aspartic acid	0.34
Aspartic acid	0.26	Aspartic acid	0.60	Glutamic acid	1.42	Glutamic acid	0.73
Glutamic acid	0.68	Glutamic acid	0.62	Glutamine	0.17	Glutamine	0.17
Glutamine	0.18	Glutamine	0.47	Lysine	3.39	Lysine	0.42
Homoserine	0.23	Homoserine	0.67	Phenylalanine	2.53	Phenylalanine	0.09
Isoleucine	0.12	Isoleucine	0.59	Proline	4.69	Proline	0.19
Leucine	0.09	Leucine	0.60	Tryptophan	0.56	Tryptophan	0.07
Serine	<0.2	Serine	0.69	Tyrosine	1.73	Tyrosine	0.28
Threonine	0.21	Threonine	0.65	Valine	2.37	Valine	0.30
Tryptophan	0.05	Tryptophan	0.40			Alanine	0.23
Valine	0.25	Valine	0.67			Arginine	0.31
Arginine	0.31					Histidine	0.63
Histidine	0.58					Homoserine	0.26
Lysine	0.05					Isoleucine	0.16
Methionine	0.35					Leucine	0.09
Phenylalanine	0.07					Methionine	0.33
Proline	0.17					Serine	<0.2
Tyrosine	0.25					Threonine	0.25
				Antioxidants			
Curcumin	1.99			Chlorogenic acid	9.49	Chlorogenic acid	0.69
Glutathione reduced	<0.2			Curcumin	23.86	Curcumin	2.38
				Fatty acids			
Linolenic acid	0.60			Linoleic acid	<0.2	Linolenic acid	<0.2
				Linolenic acid	0.84		
				Nucleobase/side/tide			
Adenosine	<0.2			Adenosine	3.58	Adenosine	<0.2
Guanosine	<0.2			Guanosine	2.02	Guanosine	<0.2
Uridine	<0.2					Cytidine	<0.2
						Uridine	<0.2
				Organic acids/phenolics			
Succinic acid	<0.2			Ascorbic acid	5.91	Citric acid	1.52
				Malic acid	0.25		
				Succinic acid	0.31		
				Sugar/alcohols			
Maltose	0.64	Maltose	0.79	Fructose	0.51	Fructose	0.25
Raffinose	0.24	Raffinose	0.34	Maltose	1.68	Maltose	1.41
Sucrose	0.74	Sucrose	0.80	Ribitol/Xylitol	1.12	Ribitol/Xylitol	0.89
Fructose	0.43			Sucrose	1.77	Sucrose	1.63
Glucose/Galactose	0.44			Trehalose	<0.2	Trehalose	<0.2
Mannose	0.39					Glucose/Galactose	0.41
Trehalose	<0.2					Mannose	0.31

Experimental conditions: SA—200 mg/L Triton™ X-100 solution; $Cu(OH)_2$ NMs—100 mg/L $Cu(OH)_2$ NMs (as Cu content) in SA; Cu IONs—0.1 mg/L $CuSO_4 \cdot 5H_2O$ (as Cu content) in SA; MoO_3 NMs—100 mg/L MoO_3 NMs (as Mo content) in SA; and Mo IONs—35 mg/L $Na_2MoO_4 \cdot 2H_2O$ (as Mo content) in SA.

Table S9. Perturbed pathway analysis in wheat plant tissues after one-week foliar exposures to 100 mg/L of Cu $(OH)_2$ and MoO_3 NMs suspensions.

Disturbed pathways	*p* value	Impact factor
Leaf-$Cu(OH)_2$ NPs		
Starch and sucrose metabolism	9.304×10^{-6}	0.50
Galactose metabolism	9.610×10^{-6}	0.13
Phenylalanine metabolism	2.276×10^{-5}	0.42
Tryptophan metabolism	7.458×10^{-5}	0.17
Aminoacyl-tRNA biosynthesis	9.267×10^{-5}	0.11
alpha-Linolenic acid metabolism	1.186×10^{-4}	0.11
Glycine, serine and threonine metabolism	1.784×10^{-4}	0.58
Tyrosine metabolism	2.315×10^{-4}	0.17
Isoquinoline alkaloid biosynthesis	2.315×10^{-4}	0.41
Alanine, aspartate and glutamate metabolism	2.447×10^{-4}	0.64
Cysteine and methionine metabolism	1.074×10^{-3}	0.18
Glyoxylate and dicarboxylate metabolism	1.749×10^{-3}	0.18
Arginine biosynthesis	2.723×10^{-3}	0.40
Arginine and proline metabolism	3.469×10^{-3}	0.38
Citrate cycle (TCA cycle)	1.341×10^{-2}	0.16
Stilbenoid, diarylheptanoid and gingerol biosynthesis	1.387×10^{-2}	0.11
Linoleic acid metabolism	3.412×10^{-2}	1.00
Leaf-MoO_3 NPs		
Cysteine and methionine metabolism	1.368×10^{-6}	0.13
Linoleic acid metabolism	1.647×10^{-5}	1.00
Aminoacyl-tRNA biosynthesis	1.046×10^{-4}	0.11
Arginine and proline metabolism	7.264×10^{-4}	0.38
Arginine biosynthesis	8.208×10^{-4}	0.40
Alanine, aspartate and glutamate metabolism	2.776×10^{-3}	0.64
Citrate cycle (TCA cycle)	3.414×10^{-3}	0.16
Glutathione metabolism	4.792×10^{-3}	0.40
Stilbenoid, diarylheptanoid and gingerol biosynthesis	5.057×10^{-3}	0.11
Glycine, serine and threonine metabolism	1.268×10^{-2}	0.37
Starch and sucrose metabolism	1.563×10^{-2}	0.50
Galactose metabolism	1.571×10^{-2}	0.13
Phenylalanine metabolism	4.289×10^{-2}	0.42
Root-$Cu(OH)_2$ NPs		
Tryptophan metabolism	3.790×10^{-5}	0.17
Aminoacyl-tRNA biosynthesis	4.650×10^{-5}	0.11
Starch and sucrose metabolism	6.070×10^{-5}	0.50
Alanine, aspartate and glutamate metabolism	6.160×10^{-5}	0.64
Phenylalanine metabolism	1.812×10^{-4}	0.42
alpha-Linolenic acid metabolism	3.050×10^{-4}	0.11
Galactose metabolism	3.737×10^{-4}	0.13
Glycine, serine and threonine metabolism	7.316×10^{-4}	0.37
Cysteine and methionine metabolism	1.596×10^{-3}	0.14
Isoquinoline alkaloid biosynthesis	1.714×10^{-3}	0.41
Valine, leucine and isoleucine biosynthesis	$1.825 \times 10^{-}$	0.11
Glutathione metabolism	3.200×10^{-3}	0.40
Arginine biosynthesis	6.217×10^{-3}	0.40
Arginine and proline metabolism	8.812×10^{-3}	0.38
Citrate cycle (TCA cycle)	1.808×10^{-2}	0.16

Linoleic acid metabolism	3.356×10^{-2}	1.00
Tyrosine metabolism	4.528×10^{-2}	0.17
Root-MoO_3 NPs		
Stilbenoid, diarylheptanoid and gingerol biosynthesis	1.685×10^{-4}	0.11
Linoleic acid metabolism	1.838×10^{-4}	1.00
Alanine, aspartate and glutamate metabolism	2.371×10^{-4}	0.64
Starch and sucrose metabolism	4.435×10^{-4}	0.50
Aminoacyl-tRNA biosynthesis	1.447×10^{-3}	0.11
Arginine biosynthesis	2.126×10^{-3}	0.40
Phenylalanine metabolism	1.133×10^{-2}	0.42
Arginine and proline metabolism	3.609×10^{-2}	0.38
Tryptophan metabolism	4.235×10^{-2}	0.17

Table S10. Perturbation pathway analysis in wheat plant tissues after one-week foliar exposures to 0.1 mg/L of $CuSO_4 \cdot 5H_2O$ $_4$ and 35 mg/L of $Na_2MoO_4 \cdot 2H_2O$ solutions.

Disturbed pathways	*p* value	Impact factor
Leaf-$CuSO_4$		
Glycine, serine and threonine metabolism	3.071×10^{-6}	0.58
Glycolysis / Gluconeogenesis	1.096×10^{-5}	0.12
Pyruvate metabolism	1.096×10^{-5}	0.17
Galactose metabolism	1.784×10^{-4}	0.13
Cysteine and methionine metabolism	9.304×10^{-4}	0.14
Starch and sucrose metabolism	1.279×10^{-3}	0.50
Alanine, aspartate and glutamate metabolism	3.173×10^{-3}	0.64
Tyrosine metabolism	4.417×10^{-3}	0.17
Glutathione metabolism	5.650×10^{-3}	0.47
Arginine biosynthesis	1.886×10^{-2}	0.40
Aminoacyl-tRNA biosynthesis	3.114×10^{-2}	0.11
Tryptophan metabolism	3.147×10^{-2}	0.17
Leaf-Na_2MoO_4		
Linoleic acid metabolism	1.647×10^{-5}	1.00
Starch and sucrose metabolism	2.665×10^{-5}	0.50
Phenylalanine metabolism	4.338×10^{-5}	0.42
alpha-Linolenic acid metabolism	1.186×10^{-4}	0.11
Aminoacyl-tRNA biosynthesis	1.632×10^{-4}	0.11
Arginine biosynthesis	1.789×10^{-4}	0.40
Alanine, aspartate and glutamate metabolism	1.985×10^{-4}	0.64
Tyrosine metabolism	3.252×10^{-4}	0.17
Isoquinoline alkaloid biosynthesis	3.252×10^{-4}	0.41
Galactose metabolism	3.571×10^{-4}	0.13
Cysteine and methionine metabolism	5.173×10^{-4}	0.13
Arginine and proline metabolism	5.208×10^{-4}	0.38
Glycine, serine and threonine metabolism	8.845×10^{-4}	0.37
Citrate cycle (TCA cycle)	1.078×10^{-2}	0.16
Stilbenoid, diarylheptanoid and gingerol biosynthesis	2.551×10^{-2}	0.11
Tryptophan metabolism	4.350×10^{-2}	0.17
Root-$CuSO_4$		
Alanine, aspartate and glutamate metabolism	4.552×10^{-4}	0.64
Tryptophan metabolism	3.247×10^{-3}	0.17
Aminoacyl-tRNA biosynthesis	1.392×10^{-2}	0.11

Galactose metabolism	1.684×10^{-2}	0.13
Glycine, serine and threonine metabolism	2.462×10^{-2}	0.37
Starch and sucrose metabolism	2.557×10^{-2}	0.50
Arginine biosynthesis	2.686×10^{-2}	0.40
Root-Na_2MoO_4		
Starch and sucrose metabolism	3.005×10^{-5}	0.50
Tryptophan metabolism	4.445×10^{-5}	0.17
Aminoacyl-tRNA biosynthesis	5.041×10^{-5}	0.11
Phenylalanine metabolism	7.764×10^{-5}	0.42
Alanine, aspartate and glutamate metabolism	1.392×10^{-4}	0.64
alpha-Linolenic acid metabolism	3.050×10^{-4}	0.11
Glycine, serine and threonine metabolism	4.624×10^{-4}	0.37
Cysteine and methionine metabolism	1.961×10^{-3}	0.14
Valine, leucine and isoleucine biosynthesis	2.326×10^{-3}	0.11
Galactose metabolism	2.497×10^{-3}	0.13
Isoquinoline alkaloid biosynthesis	2.523×10^{-3}	0.41
Arginine biosynthesis	8.120×10^{-3}	0.40
Citrate cycle (TCA cycle)	8.441×10^{-3}	0.16
Arginine and proline metabolism	1.074×10^{-2}	0.38
Tyrosine metabolism	4.929×10^{-2}	0.17

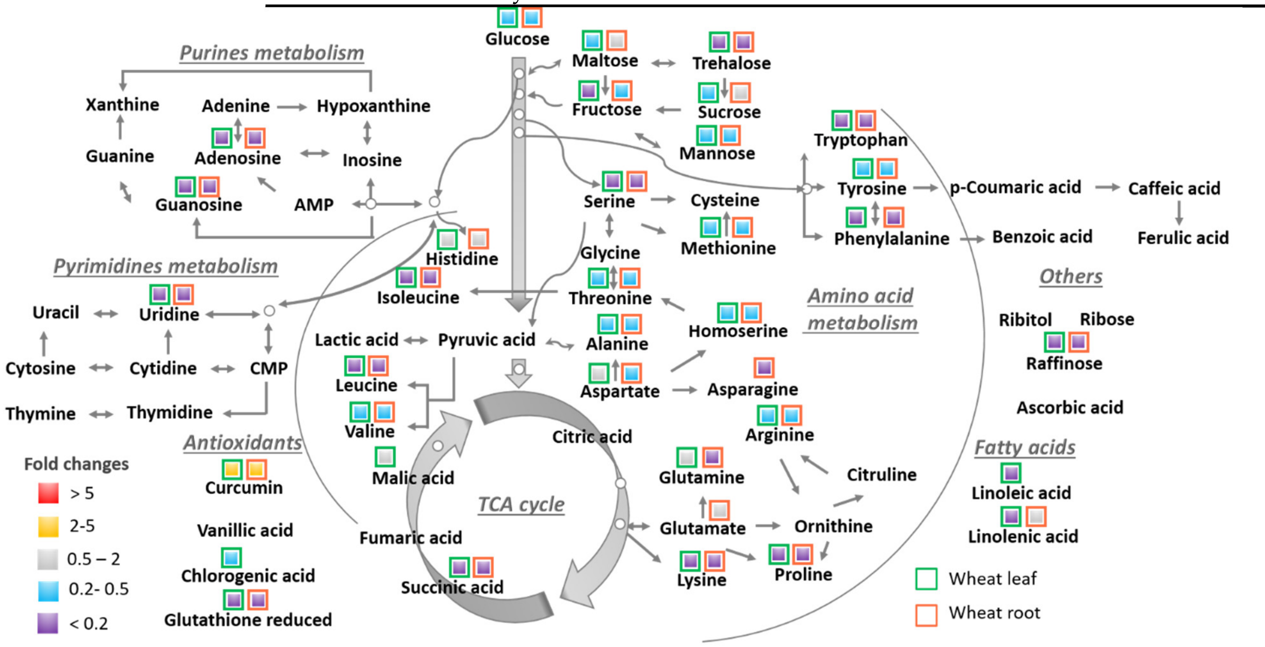

Figure S8. Significant changes in metabolite pathway of wheat after one-week foliar exposure to 100 mg/L of $Cu(OH)_2$ NMs suspension. The color scale indicates the fold changes compared with the surfactant-only solution. The border of the box indicates whether metabolite changes were in wheat leaves (in green) or roots (in red).

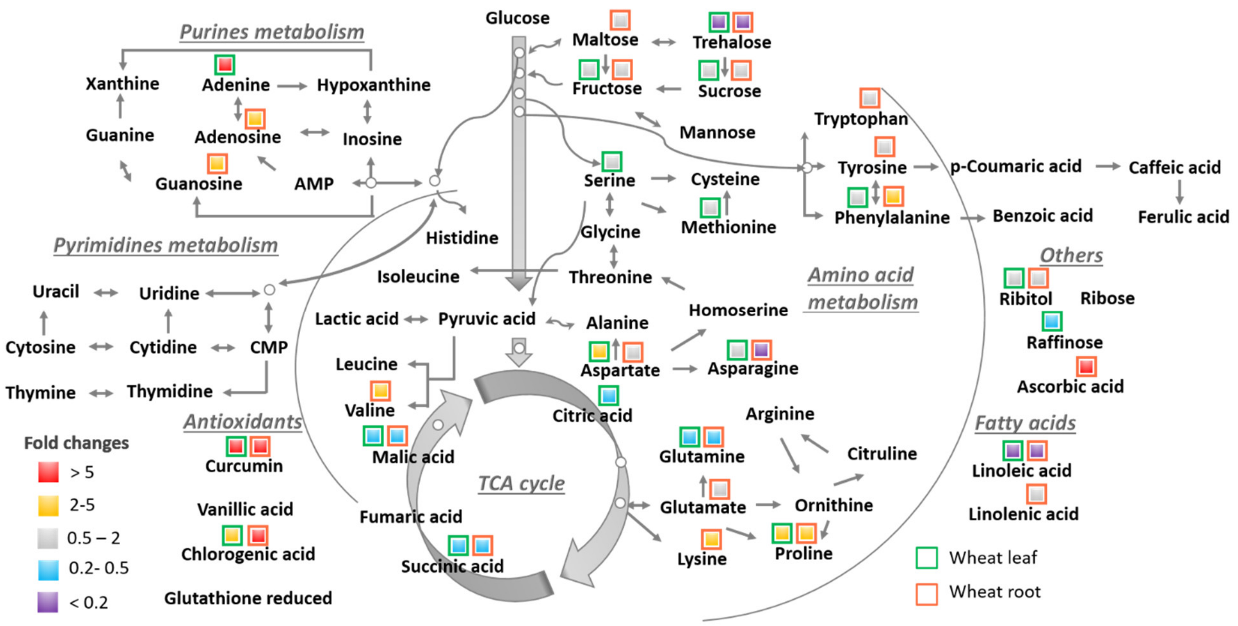

Figure S9. Significant changes in metabolite pathway of wheat after one-week foliar exposure to 100 mg/L of MoO_3 NMs suspension. The color scale indicates the fold changes compared with the surfactant-only solution. The border of the box indicates whether metabolite changes were in wheat leaves (in green) or roots (in red).

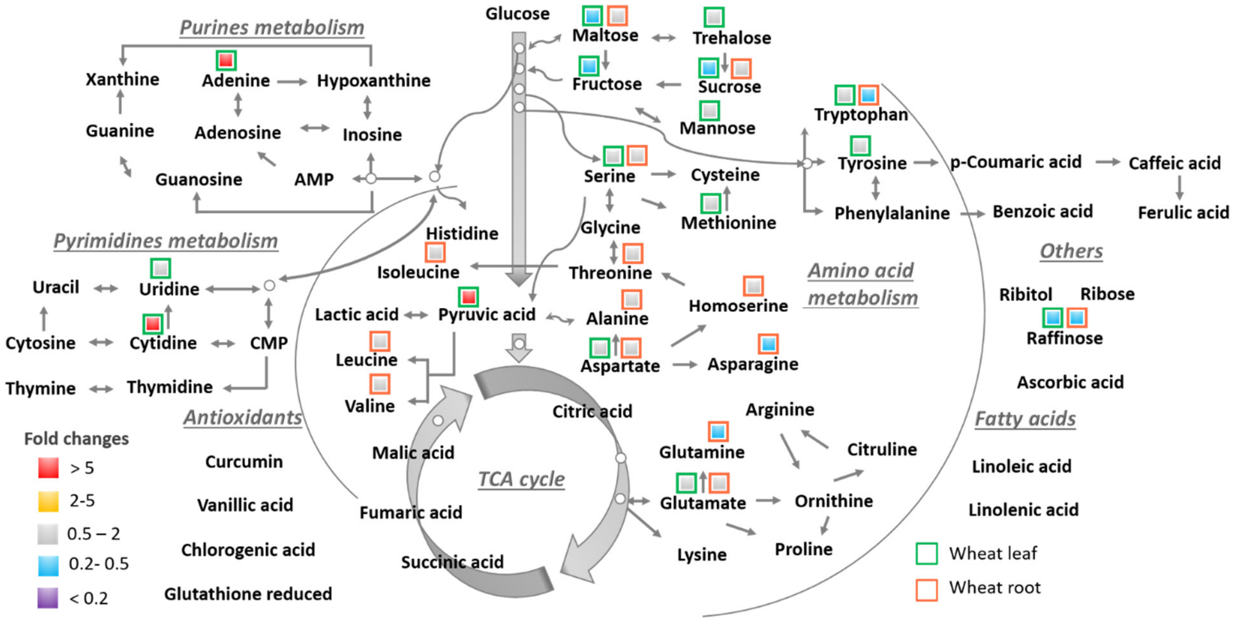

Figure S10. Significant changes in metabolic pathways of wheat after one-week foliar exposure to 0.1 mg/L of $CuSO_4 \cdot 5H_2O$ solution. The color scale indicates the fold changes compared to DI only. The border of the box indicates whether metabolite changes were wheat leaf (in green) or root (in red) specific.

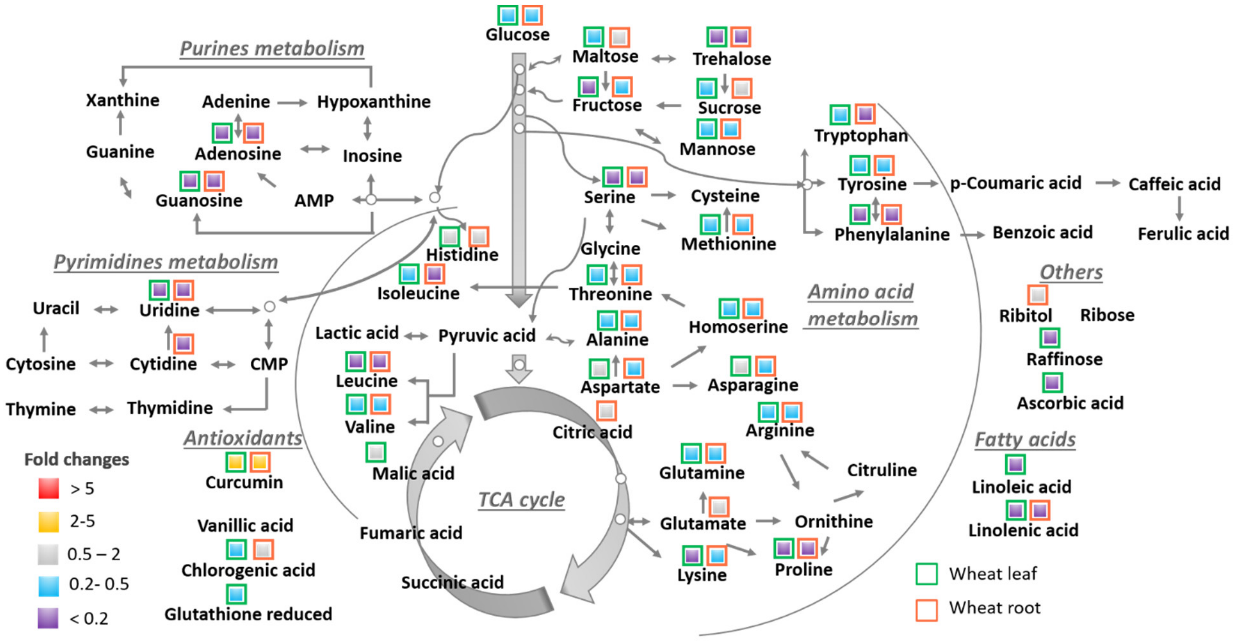

Figure S11. Significant changes in metabolic pathways of wheat after one-week foliar exposure to 35 mg/L of $Na_2MoO_4 \cdot 2H_2O$ solution. The color scale indicates the fold changes compared to DI only. The border of the box indicates whether metabolite changes were wheat leaf (in green) or root (in red) specific.

Table S11. Venn diagram results of dysregulated metabolites in wheat after one-week foliar exposure to 100 mg/L of $Cu(OH)_2$ and MoO_3 NMs suspensions.

Conditions	Shared Sig. Metabolites
a-L; c-L; a-R; c-R	Trehalose
	Succinic acid
	Glutamine
	Sucrose
	Curcumin
	Proline
	Fructose
	Phenylalanine
	Aspartic acid
a-L; c-L; a-R	Raffinose
	Methionine
	Serine
a-L; c-L; c-R	Linoleic acid
	Malic acid
	Chlorogenic acid
a-L; a-R; c-R	Tyrosine
	Valine
	Guanosine
	Tryptophan
	Maltose
	Linolenic acid
	Adenosine
	Lysine
c-L; a-R; c-R	Asparagine
a-L; a-R	Glucose/Galactose
	Alanine
	Uridine
	Threonine
	Glutathione reduced
	Histidine
	Isoleucine
	Arginine
	Leucine
	Homoserine
	Mannose
c-L; c-R	Ribitol/Xylitol
a-R c-R	Glutamic acid
c-L	Citric acid
	Adenine
c-R	Ascorbic acid

Experimental conditions: a-L—metabolites that extracted from wheat leaves after exposing to the 100 mg/L of Cu $(OH)_2$ NMs suspension; c-L—metabolites that extracted from wheat leaves after exposing to the 100 mg/L of MoO_3 NMs suspensions; a-R—metabolites that extracted from wheat roots after exposing to the 100 mg/L of Cu $(OH)_2$ NMs suspension; c-R—metabolites that extracted from wheat roots after exposing to the 100 mg/L of MoO_3 NMs suspension.

Table S12. Venn diagram results of perturbed metabolic pathways in wheat after one-week foliar exposure to 100 mg/L of Cu $(OH)_2$ and MoO_3 NMs suspensions.

**Conditions**	**Sig. Disturbed pathways**
a-L; c-L; a-R; c-R	Alanine, aspartate and glutamate metabolism
	Arginine biosynthesis
	Linoleic acid metabolism
	Starch and sucrose metabolism
	Aminoacyl-tRNA biosynthesis
	Phenylalanine metabolism
	Arginine and proline metabolism
a-L; c-L; a-R	Citrate cycle (TCA cycle)
	Cysteine and methionine metabolism
	Glycine, serine and threonine metabolism
	Galactose metabolism
a-L; c-L; c-R	Stilbenoid, diarylheptanoid and gingerol biosynthesis
a-L; a-R; c-R	Tryptophan metabolism
a-L; a-R	alpha-Linolenic acid metabolism
	Isoquinoline alkaloid biosynthesis
	Tyrosine metabolism
c-L; a-R	Glutathione metabolism
a-L	Glyoxylate and dicarboxylate metabolism
a-R	Valine, leucine and isoleucine biosynthesis

Experimental conditions: a-L—metabolites that extracted from wheat leaves after exposing to the 100 mg/L of Cu $(OH)_2$ NMs suspension; c-L—metabolites that extracted from wheat leaves after exposing to the 100 mg/L of MoO_3 NMs suspensions; a-R—metabolites that extracted from wheat roots after exposing to the 100 mg/L of Cu $(OH)_2$ NMs suspension; c-R—metabolites that extracted from wheat roots after exposing to the 100 mg/L of MoO_3 NMs suspensions.
